# LP340, a novel histone deacetylase inhibitor, decreases liver injury and fibrosis in mice: role of oxidative stress and microRNA-23a

**DOI:** 10.3389/fphar.2024.1386238

**Published:** 2024-05-17

**Authors:** Devadoss J. Samuvel, John J. Lemasters, C. James Chou, Zhi Zhong

**Affiliations:** ^1^ Departments of Drug Discovery and Biomedical Sciences, Charleston, SC, United States; ^2^ Biochemistry and Molecular Biology, Medical University of South Carolina, Charleston, SC, United States; ^3^ Lydex Pharmaceuticals, Mount Pleasant, SC, United States

**Keywords:** histone deacetylase, liver fibrosis, microRNA-23a, oxidative stress, SnoN, TGFβ

## Abstract

Effective therapy for liver fibrosis is lacking. Here, we examined whether LP340, the lead candidate of a new-generation of hydrazide-based HDAC1,2,3 inhibitors (HDACi), decreases liver fibrosis. Liver fibrosis was induced by CCl_4_ treatment and bile duct ligation (BDL) in mice. At 6 weeks after CCl_4_, serum alanine aminotransferase increased, and necrotic cell death and leukocyte infiltration occurred in the liver. Tumor necrosis factor-α and myeloperoxidase markedly increased, indicating inflammation. After 6 weeks, α-smooth muscle actin (αSMA) and collagen-1 expression increased by 80% and 575%, respectively, indicating hepatic stellate cell (HSC) activation and fibrogenesis. Fibrosis detected by trichrome and Sirius-red staining occurred primarily in pericentral regions with some bridging fibrosis in liver sections. 4-Hydroxynonenal adducts (indicator of oxidative stress), profibrotic cytokine transforming growth factor-β (TGFβ), and TGFβ downstream signaling molecules phospho-Smad2/3 also markedly increased. LP340 attenuated indices of liver injury, inflammation, and fibrosis markedly. Moreover, Ski-related novel protein-N (SnoN), an endogenous inhibitor of TGFβ signaling, decreased, whereas SnoN expression suppressor microRNA-23a (miR23a) increased markedly. LP340 (0.05 mg/kg, ig., daily during the last 2 weeks of CCl_4_ treatment) decreased 4-hydroxynonenal adducts and miR23a production, blunted SnoN decreases, and inhibited the TGFβ/Smad signaling. By contrast, LP340 had no effect on matrix metalloproteinase-9 expression. LP340 increased histone-3 acetylation but not tubulin acetylation, indicating that LP340 inhibited Class-I but not Class-II HDAC *in vivo*. After BDL, focal necrosis, inflammation, ductular reactions, and portal and bridging fibrosis occurred at 2 weeks, and αSMA and collagen-1 expression increased by 256% and 560%, respectively. LP340 attenuated liver injury, ductular reactions, inflammation, and liver fibrosis. LP340 also decreased 4-hydroxynonenal adducts and miR23a production, prevented SnoN decreases, and inhibited the TGFβ/Smad signaling after BDL. *In vitro*, LP340 inhibited immortal human hepatic stellate cells (hTERT-HSC) activation in culture (αSMA and collagen-1 expression) as well as miR23a production, demonstrating its direct inhibitory effects on HSC. In conclusions, LP340 is a promising therapy for both portal and pericentral liver fibrosis, and it works by inhibiting oxidative stress and decreasing miR23a.

## 1 Introduction

Liver fibrosis is the excessive accumulation of extracellular matrix (ECM) including collagen in the liver, which occurs in patients with many chronic liver diseases (Pinzani, 1999; Bataller and Brenner, 2005). Depending on the background chronic liver disease, liver fibrosis may begin primarily around portal tracts (portal fibrosis), such as in chronic cholestatic liver disease and viral hepatitis, or occurs initially in pericentral and perisinusoidal areas (pericentral/perivenular fibrosis), such as in alcohol-associated liver disease (Pinzani, 1999; Bataller and Brenner, 2005). As hepatic fibrosis advances, bridging fibrosis is formed and ultimately cirrhosis occurs (Pinzani, 1999; Bataller and Brenner, 2005). In its advanced stage, liver cirrhosis, parenchymal cells are replaced by collagen fibers, liver architecture becomes distorted, and hepatic blood flow is disturbed, leading to portal hypertension, chronic liver failure, severe complications, and death (Crawford, 2002; Rahimi and Rockey, 2013). Liver fibrosis/cirrhosis affects >100 million people and represents one of the most common causes of death in adults in the world (Friedman, 2003; Asrani et al., 2019). Moreover, 60%–90% of cases of hepatocellular carcinoma (HCC), a highly malignant tumor, arise on a background of liver fibrosis (Scaglione et al., 2015).

Although the most effective anti-fibrotic therapies are those targeting the underlying diseases causing fibrogenesis, such as antiviral therapy for viral hepatitis and iron chelation for hemochromatosis (Powell and Kerr, 1970; Poynard et al., 2002; Hadziyannis et al., 2003), therapy for many liver diseases is still lacking, working only in certain patient populations, or incompletely effective. Moreover, onset of many liver diseases and the subsequent development of liver fibrosis is often insidious. In the U.S, majority of liver disease patients do not know that they have liver disease until advanced fibrosis occurs, which substantially delays the treatment of the underlying diseases (Scaglione et al., 2015). Despite extensive studies, FDA-approved pharmaceutical therapy for fibrosis is still unavailable. The only clinically proven treatment for cirrhosis is liver transplantation (LT), whereas the availability of LT is very low due to the severe shortage of donor livers (Meirelles Junior et al., 2015). Thus, blockade of common profibrogenic and proinflammatory pathways and/or stimulation of resolution of fibrosis represents possible alternative therapeutic approaches. No doubt, development of effective antifibrotic therapy is an urgent need for medicine.

Histone deacetylases (HDACs) are a group of epigenetic enzymes that catalyze histone deacetylation, which subsequently alters gene expression (Kuo and Allis, 1998). Histone deacetylation is also strongly associated with dysregulated expression of microRNAs, which can affect numerous biological and pathological processes (Suzuki et al., 2013; Soliman et al., 2018; Ramzan et al., 2021). In addition to deacetylating histones, HDACs also remove acetyl moieties from lysine residues on non-histone proteins, thus affecting their activation, localization, function, and degradation (Kiernan et al., 2003; Glozak et al., 2005). Eighteen isoforms of HDAC exist in mammals, belonging to 4 classes (Hammond et al., 2017; Yoon et al., 2019). HDACs regulate many biological and pathological processes, including cell death, proliferation, inflammation, fibrosis, and cancer (Shao et al., 2004; Tao et al., 2014; Li et al., 2020). In recent years, accumulated evidence suggests that HDACs stimulate fibrogenesis in several organs, such as the heart, lung, kidney, and liver (Van Beneden et al., 2013; Chen et al., 2015; Moran-Salvador and Mann, 2017; Yoon et al., 2019). As global levels of acetylation of histones H3 and H4 progressively decrease, hepatic stellate cell (HSC) activation, a key step of liver fibrosis, occurs (Chen et al., 2015). Inhibition of HDAC1-3 (Class I HDAC) suppresses HSC activation and induces apoptosis and autophagic cell death of activated HSC (Liu et al., 2013; Shaker et al., 2013). Some studies show that knockdown of HDAC4, but not HDAC5 or HDAC6 (Class II HDAC), partially hinders HSC activation through induction of microRNA-29 (Mannaerts et al., 2013). Some HDAC inhibitors (HDACi) [e.g., trichostatin A (TSA) and suberoylanilide hydroxamic acid (SAHA)] have been shown to decrease fibrotic responses *in vitro* and *in vivo* (Mannaerts et al., 2010; Shaker et al., 2013; Van Beneden et al., 2013; Chen et al., 2015). However, successful clinical trials of HDACi against liver fibrosis have not been reported. Possibly, different subtypes of HDAC exert different effects on the development and resolution of fibrosis. Therefore, isozyme selective HDACi may be needed for prevention/treatment of fibrosis. Moreover, all HDACi currently on the market are for cancer therapy, and all have poor *in vivo* pharmacokinetics (PK), low HDAC isozyme selectivity, and long-term safety concerns regarding potential mutagenicity (Shen and Kozikowski, 2016). Therefore, more selective, potent, and safe HDACi are needed for assessment in treating liver fibrosis.

Recently, we described a new generation of hydrazide-based HDACi that inhibit HDAC1,2,3 both allosterically and competitively. These HDACi have substantially higher potency, excellent PK properties, and lower toxicity (McClure et al., 2016; Li et al., 2018). Moreover, we showed recently that LP342, one of the lead compounds of new class of HDACi, protects against hepatic ischemia/reperfusion injury (Samuvel et al., 2023). In this study, we explored whether LP340, another lead compound, decreases liver fibrosis and examined potential mechanisms of its protection.

## 2 Materials and methods

### 2.1 Materials

The sources of all chemicals, antibodies, and other reagents are listed in Supplementary Table S1 in “Supplementary Material.”

### 2.2 Synthesis of LP340

The molecular structure, synthesis, purification, and characterization of LP340 are described elsewhere (Jiang et al., 2022).

### 2.3 Animals

**FIGURE 1:**
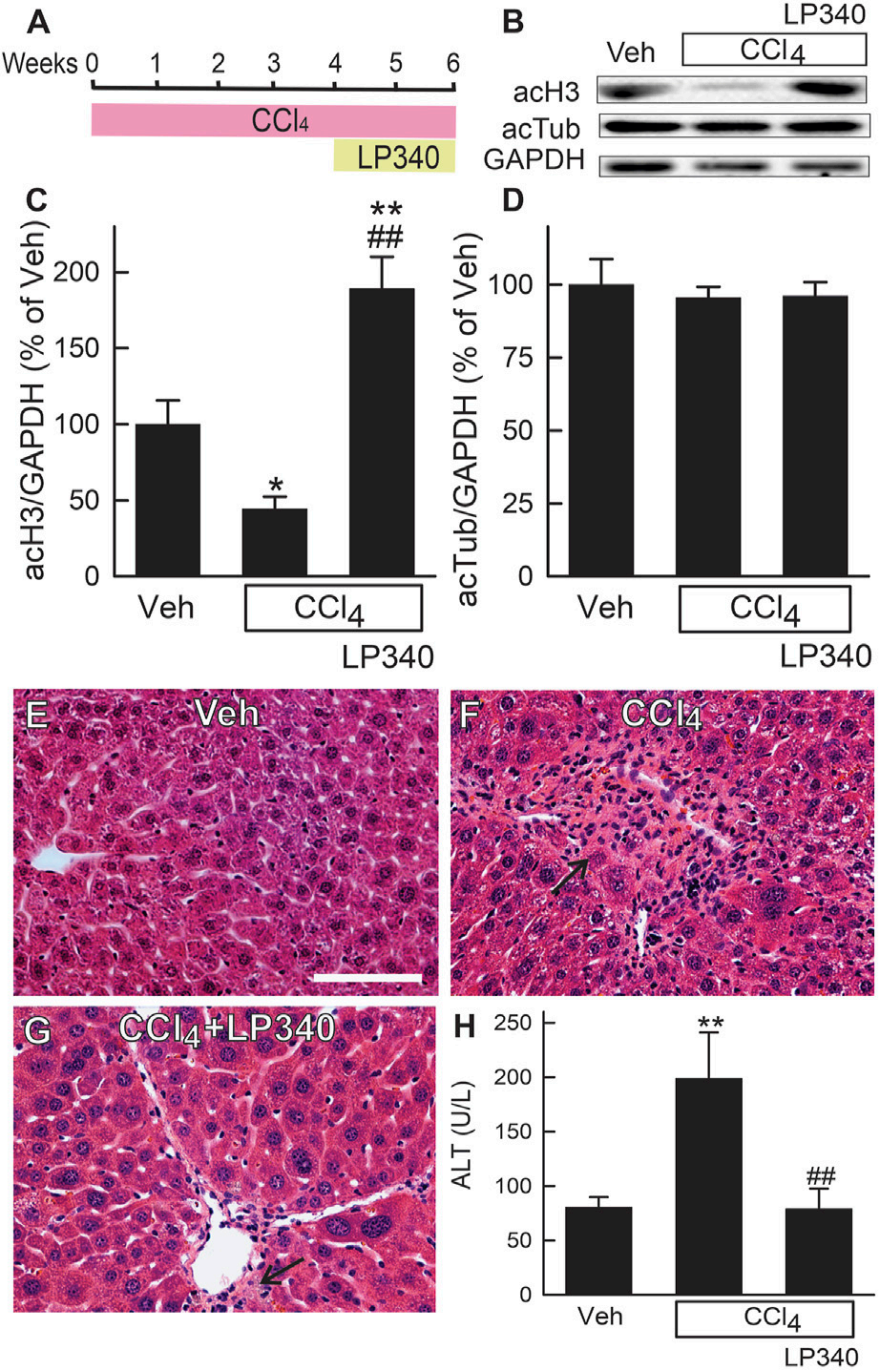
LP340 increases histone-3 acetylation and decreases alanine aminotransferase release and necrosis after CCl_4_ treatment. Male mice were injected with CCl_4_ (1:3 dilution in corn oil; 1.0 µL of dilution/g mouse, *i.p*.) or equal volume of corn oil once every 3 days for 6 weeks. LP340 (0.05 mg/kg, ig daily) or equal volume of vehicle (Veh) was administered during the last 2 weeks of CCl_4_ treatment. Blood and liver were collected after 6 weeks of CCl_4_ or corn oil treatment. **(A)** regimen of CCl_4_ and LP340 treatment. **(B)** representative immunoblots for acetylated histone-3 (acH3), acetylated tubulin (acTub), and housekeeping protein GAPDH; **(C, D)**, quantification of acH3 and acTub immunoblots by densitometry. **(E–G)** representative images of liver histology. Bar is 50 µm. **(H)** serum ALT. *, *p* < 0.05 vs. vehicle; **, *p* < 0.01 vs. vehicle; ##, *p* < 0.01 vs. CCl_4_ without LP340. Data are means ± SEM (n = 3–4/group).

Liver fibrosis was induced in mice by bile duct ligation (BDL) and carbon tetrachloride (CCl_4_) treatment, respectively (Trams and Symons, 1957; Rehman et al., 2008; Rehman et al., 2016). For BDL, male C57Bl/6 mice (8–9 weeks, Jackson Laboratory, Bar Harbor, ME) underwent a midline abdominal incision under isoflurane (2%–3%) anesthesia. The common bile duct was located, double ligated near the liver with 6-0 nylon suture, and transected between ligatures. For sham operation, the abdomen was opened under isoflurane anesthesia and then closed without BDL. During surgery, body temperature was maintained at ∼37°C with warming lamps. Mice were gavaged with LP340 (0.05 mg/kg) or equal volume of vehicle (0.5% DMSO, 0.5% hydroxypropyl methyl cellulose) at 2 h after surgery and once daily afterwards for 2 weeks at which time the mice were euthanized. To induce liver fibrosis with CCl_4_, male mice were injected with CCl_4_ (1:3 dilution in corn oil; 1.0 µL of dilution/g mouse, *i.p*.) or an equal volume of corn oil once every 3 days for up to 6 weeks. Since a previous study showed that female mice are more tolerant to CCl_4_ hepatotoxicity (Zhang et al., 2012), female mice were treated with 1.5 µL of the CCl_4_ dilution/g mouse, *i.p*.). Both male and female mice were treated with LP340 (0.05 mg/kg, *i.g.*) or equal volume of vehicle once daily during the last 2 weeks of CCl_4_ treatment (Figure 1A). Mice were euthanized after 6 weeks of CCl_4_ treatment. All animals were given humane care in compliance with institutional guidelines using protocols approved by the Institutional Animal Care and Use Committee of the Medical University of South Carolina (protocol number ARC# 2018-00641).

### 2.4 Culture of immortal human hepatic stellate cells

Activation of HSC is the key step in liver fibrosis. Therefore, we examined if LP340 suppresses HSC activation *in vitro*. Immortal human HSC (hTERT-HSC), a cell line that is often used to study the cell biology of human HSC *in vitro* (Schnabl et al., 2002), were cultured in DMEM high glucose medium (Supplementary Table S1) supplemented with 10% fetal bovine serum (FBS) at 5% CO_2_ and 37°C for 24 h to reach ∼70% confluence and then changed to the DMEM medium with 0.5% FBS and with or without LP340 (0.1 and 0.3 µM) for another 48 h. hTERT-HSC were then collected, lysed with ice-cold lysis buffer (Subedi et al., 2019), and lysates were used for immunoblotting or microRNA-23a (miR23a) measurement.

### 2.5 Serum alanine aminotransferase measurement

At the end of experiments, blood and liver were collected from mice under ketamine/xylazine anesthesia (90 mg/kg and 10 mg/kg, *i.p*.). Blood was collected by puncture of the inferior vena cava, and serum was isolated and stored at −80°C until use. Serum alanine aminotransferase (ALT) activity was determined using a commercial kit (Supplementary Table S1) according to the manufacturer’s instructions.

### 2.6 Histology and immunohistochemistry

After collection, part of the liver was stored at −80°C until use later, and the other part was fixed in 10% neutralized formaldehyde for 24–48 h and then embedded in paraffin. Liver sections (5 µm) were stained with hematoxylin-eosin (H&E) for histological examination. Liver sections were also stained with Mason’s trichrome staining and Sirius red/Fast green staining to reveal liver fibrosis (Kiernan, 2015; Rehman et al., 2016).

Proliferation of cholangiocytes was detected by immunohistochemical staining of cytokeratin-19 (CK19). Liver sections were deparaffinized and rehydrated, followed by antigen retrieval using antigen unmasking solution (Supplementary Table S1) according to the manufacturer’s instructions. Liver sections were pre-blocked with 3% H_2_O_2_ in distilled water and 2% bovine serum albumin (Supplementary Table S1) in 1% phosphate buffered saline with 0.1% Tween-20 (PBS-T) for 1 h at room temperature. Liver sections were then incubated with primary antibody for CK19 from rabbit (Supplementary Table S1, 1:500 dilution) at room temperature for 1 h. After washing in PBS-T 3 times (3 min each), sections were incubated with peroxidase conjugated secondary anti-rabbit antibody using a VECTASTAIN ABC kit (Supplementary Table S1). 3,3′-Diaminobenzidine (DAB) (Supplementary Table S1) was used to detect peroxidase. The sections were then counterstained with 1/5 Harris hematoxylin solution (Supplementary Table S1) for 1 min at room temperature. Liver images were acquired using a Zeiss AX10 microscope (White Plains, NY) and 10x - 40x objective lenses.

### 2.7 Detection of microRNA-23a in liver tissue and cell lysates

miRNAs were extracted from liver tissue (100 mg) or hTERT-HSC lysates using a miRNeasy micro-Kit (Supplementary Table S1) according to the manufacturer’s instructions. cDNAs were synthesized from 10 ng RNA using a miCURY LNA RT kit (Supplementary Table S1). Customized probes for miR23a and housekeeping gene *U6* were used (Supplementary Table S1). Real-time PCR was performed using iQ™ SYBR Green Supermix (Supplementary Table S1) and a Bio-Rad CFX 96 Real time PCR System with incubations at 55°C for 5 min and 95°C for 5 min, followed by 39 cycles at 95°C for 10 s and 60°C for 1 min. The results were normalized to the expression of U6 miRNA. miR23a expression was determined by the delta-delta Ct method (Krishnasamy et al., 2016).

### 2.8 Immunoprecipitation of Smad4

Liver tissue was collected after 6 weeks of CCl_4_ treatment and homogenized in ice-cold lysis buffer (Subedi et al., 2019). The protein contents of lysates were determined using a Pierce BCA protein assay kit (Supplementary Table S1) according to the manufacturer’s instructions. Liver lysates with 500 μg protein was immunoprecipitated (IP) using a Pierce classic immunoprecipitation kit (Supplementary Table S1) with mothers against decapentaplegic homolog-4 (Smad4) antibody (5 μg, Supplementary Table S1) according to manufacturer’s instruction. Protein contents in immunoprecipitates were measured, and immunoprecipitates with equal amount of protein were loaded to each lane (Liu et al., 2015). Smad4 and Ski-related novel protein-N (SnoN) were measured by immunoblotting (IB) as described below.

### 2.9 Immunoblotting

Livers were collected at 2 weeks after BDL or sham-operation or 6 weeks after treatment with CCl_4_ or corn oil. Liver tissue was homogenized in ice-cold lysis buffer, as described above. Immunoblotting of proteins in tissue lysates, immunoprecipitates, or hTERT-HSC lysates was performed with primary antibodies specific for the proteins of interest, as described previously (Rehman et al., 2008). The sources of antibodies are shown in Supplementary Table S1. Horseradish peroxidase-conjugated secondary antibodies (Supplementary Table S1) were applied after removal of primary antibodies by washing with TBS-T solution, and detection was by chemiluminescence (Rehman et al., 2008).

### 2.10 Statistical analysis

Groups were compared using ANOVA plus Student-Newman-Keul’s *post hoc* test using *p* < 0.05 as the criterion of significance. Values are means ± SEM. Group sizes are described in figure legends.

## 3 Results

### 3.1 LP340 increases histone-3 but not tubulin acetylation in the liver

To determine if LP340 inhibits Class I HDAC *in vivo*, we assessed the acetylation status of histone-3 (substrate for Class I HDAC) in livers of male mice. After CCl_4_ treatment, acetylated histone-3 (acH3) decreased by 55% in vehicle-treated mice but increased ∼90% in LP340-treated mice (Figures 1B, C). By contrast, acetylation of tubulin (substrate for Class II HDAC) was not altered by CCl_4_ or LP340 (Figures 1B, D). These data demonstrate that LP340 inhibits Class I but not Class II HDACs *in vivo*.

### 3.2 LP340 decreases liver injury and inflammation after CCl_4_ treatment

In male mice, liver histology was normal after vehicle treatment (Figure 1E). After 6 weeks of CCl_4_ treatment, small necrotic foci with leukocyte infiltration (necro-inflammatory foci) and foci of leukocytes alone without overt necrosis developed (Figure 1F). Many necro-inflammatory foci localized in centrilobular regions, but some necro-inflammatory foci were scattering throughout the liver lobule. Cell swelling was also observed in some hepatocytes (not shown). Treatment with LP340 decreased these pathological changes (Figure 1G). ALT was measured at 6 weeks after vehicle or CCl_4_ treatment. In male mice, serum ALT was 80 U/L in vehicle-treated mice. After 6 weeks of CCl_4_ treatment, ALT increased to ∼200 U/L (Figure 1H). Treatment with LP340 during the last 2 weeks of CCl_4_ administration decreased serum ALT to ∼80 U/L, indicating an attenuation of CCl_4_-induced liver injury (Figure 1H).

**FIGURE 2:**
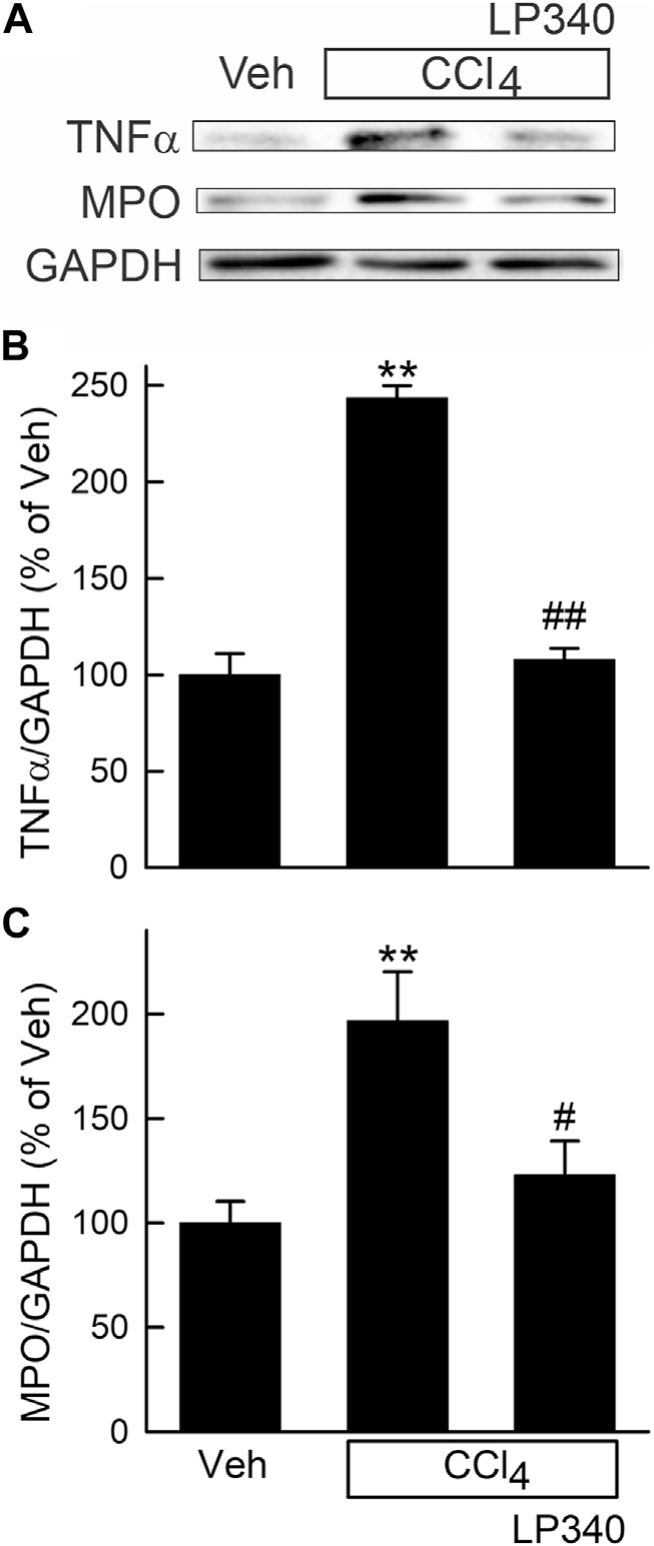
LP340 decreases inflammatory responses after CCl_4_ treatment. Male mice were treated, and livers were collected as described in Figure 1. **(A)** representative immunoblots for tumor necrosis factor-α (TNFα), myeloperoxidase (MPO), and housekeeping protein GAPDH. **(B,C)** quantification of TNFα and MPO immunoblots by densitometry. Veh, vehicle; **, *p* < 0.01 vs. vehicle; #, *p* < 0.05 vs. CCl_4_ without LP340; ##, *p* < 0.01 vs. CCl_4_ without LP340. Data are means ± SEM (n = 4/group).

Consistent with increased leukocyte infiltration in liver sections after CCl_4_ treatment, tumor necrosis factor-α (TNFα), a proinflammatory cytokine, increased ∼140% (Figures 2A, B). Myeloperoxidase (MPO), a marker of polymorphonuclear cell infiltration, also increased 98% after CCl_4_, indicating inflammation. LP340 blunted these inflammatory responses by 74% after CCl_4_ treatment (Figures 2A, C).

We also examined whether LP340 protects against liver injury in female mice after CCl_4_ treatment. Since a previous study showed that female mice are more tolerant to CCl_4_ hepatotoxicity (Zhang et al., 2012), female mice were treated with a higher dose of CCl_4._ After 6 weeks of CCl_4_, serum ALT increased to 150 U/L (Supplementary Material, Supplementary Figure S1). With LP340 treatment, serum ALT was only 88 U/L. Scattered small necro-inflammatory and inflammatory foci were again observed, which LP340 also blunted (Supplementary Figure S1).

### 3.3 LP340 decreases liver fibrosis after CCl_4_ treatment

**FIGURE 3:**
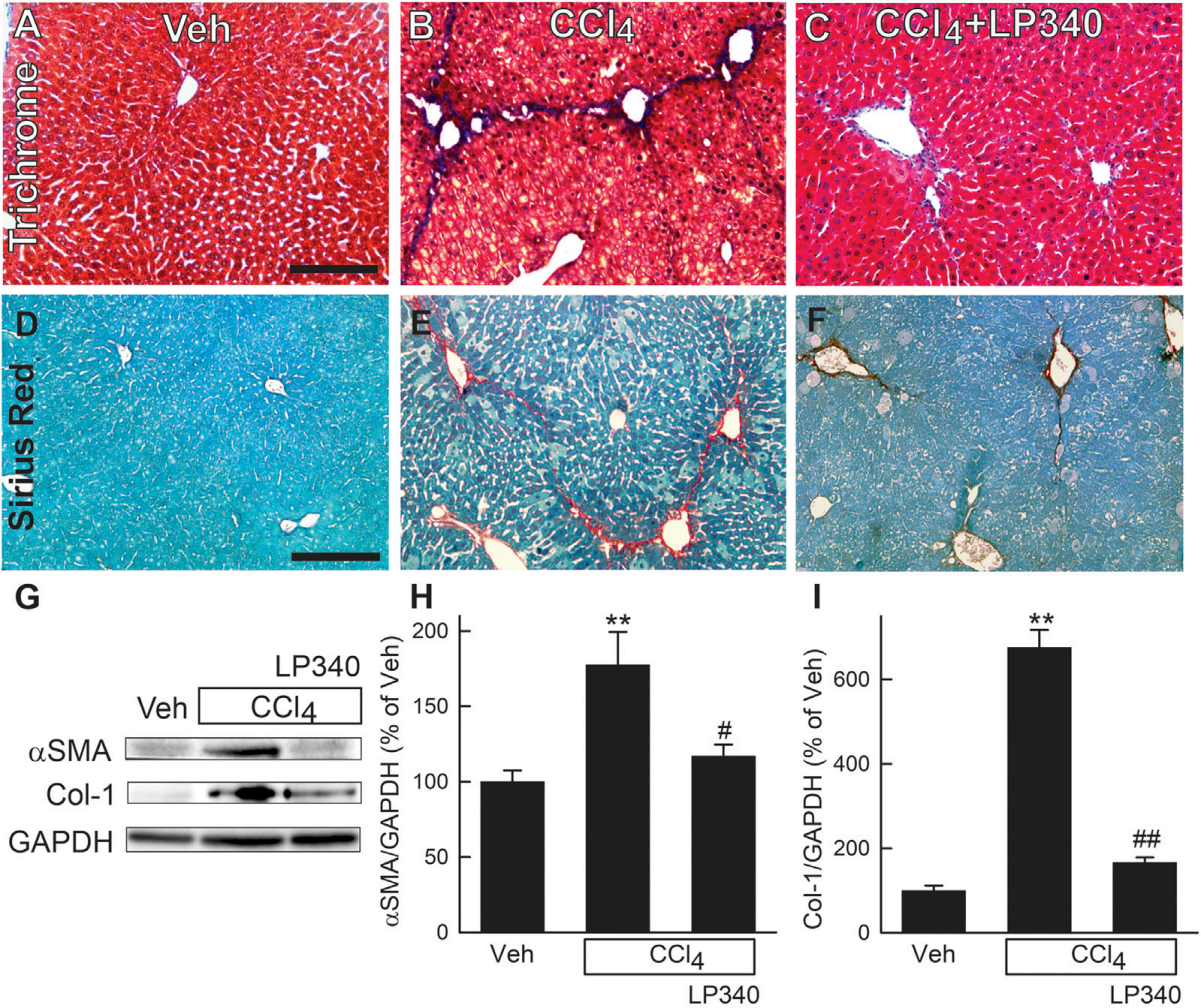
LP340 decreases liver fibrosis after CCl_4_ treatment. Male mice were treated, and livers were collected as described in Figure 1. **(A–C)** representative images of tichrome-stained liver sections. Bar is 100 μm; **(D–F)** representative images of Sirius red/Fast green-stained liver sections. Bar is 100 µm. **(G)** representative immunoblots for α-smooth muscle actin (αSMA), collagen-1 (Col-1), and housekeeping protein GAPDH. **(H,I)** quantification of αSMA and Col-1 immunoblots by densitometry. Veh, vehicle; **, *p* < 0.01 vs. vehicle; #, *p* < 0.05 vs. CCl_4_ without LP340; ##, *p* < 0.01 vs. CCl_4_ without LP340. Data are means ± SEM (n = 4/group).

Liver fibrosis was visualized by Mason’s trichrome staining and Sirius red/Fast green staining of liver sections (Kiernan, 2015; Rehman et al., 2016). After trichrome staining, collagen stained blue (Figures 3A–C), whereas after Sirius red staining, collagen was red (Figures 3D–F). In vehicle-treated male mice, collagen staining occurred only in portal tracts and around large vessels. After CCl_4_ treatment, both trichrome and Sirius red staining markedly increased, primarily in centrilobular regions and around venules. Bridging fibrosis also occurred. In some portal areas, collagen staining was also slightly increased (Figures 3B, E). LP340 decreased liver fibrosis after CCl_4_ treatment (Figures 3C, F).

Activation of HSC is a critical step in development of liver fibrosis. α-Smooth muscle actin (αSMA), an indicator of HSC activation, increased ∼80% after CCl_4_ treatment in male mice (Figures 3G, H). With LP340 treatment, αSMA increased only ∼20%. Liver fibrosis is characterized by increased formation of extracellular matrix, especially collagen. Collagen-1 expression increased 575% after CCl_4_ treatment. With LP340 treatment, collagen-1 expression increased only ∼70%. (Figures 3G, I).

In female mice, trichrome staining and collagen-1 expression also increased (Supplementary Material, Supplementary Figures S2A-S2E)), indicating that CCl_4_ treatment also caused fibrosis in females at a higher dosage. LP340 again blunted fibrosis in female mice (Supplementary Figures S2A-S2E).

### 3.4 LP340 decreased oxidative stress and inhibited TGFβ/Smad signaling after CCl_4_ treatment but did not alter matrix metalloproteinases-9 expression

**FIGURE 4:**
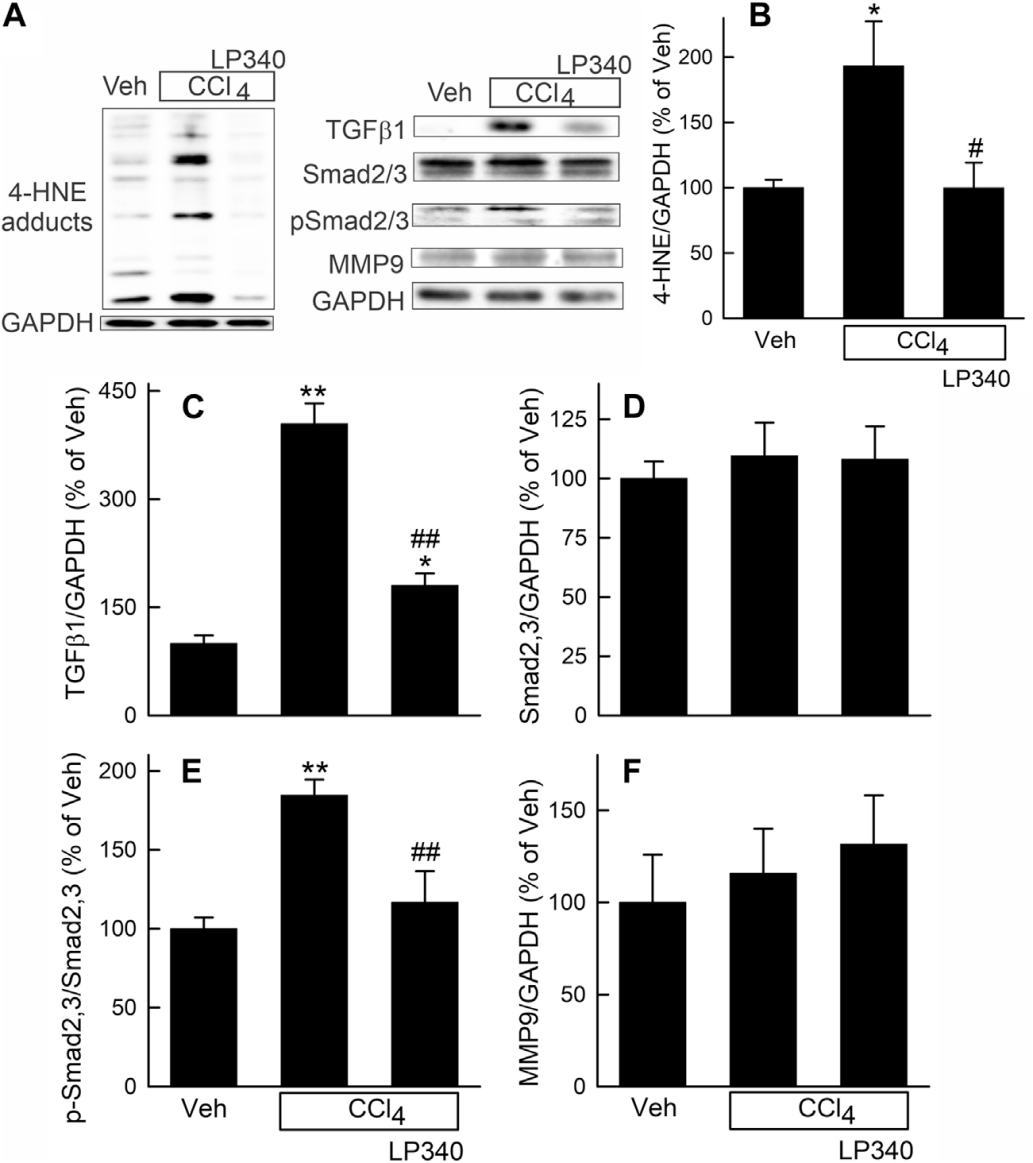
LP340 inhibits oxidative stress and TGFβ/Smad signaling but does not alter matrix metalloproteinase-9 expression after CCl_4_ treatment. Male mice were treated, and livers were collected as described in Figure 1. **(A)** representative immunoblots for 4-hydroxynonenal (4-HNE) adducts, transforming growth factor-β1 (TGFβ1), mothers against decapentaplegic homolog2,3 (Smad2,3), phospho-Smad2,3 (pSmad2,3), matrix metalloproteinase-9 (MMP9), and housekeeping protein GAPDH. **(B–F)** quantification of 4-HNE adducts, TGFβ1, Smad2,3, pSmad2,3 and MMP9 immunoblots by densitometry. Veh, vehicle; *, *p* < 0.05 vs. vehicle; **, *p* < 0.01 vs. vehicle; #, *p* < 0.05 vs. CCl_4_ without LP340; ##, *p* < 0.01 vs. CCl_4_ without LP340. Data are means ± SEM (n = 4/group).

CCl_4_ is known to cause oxidative stress, and oxidative stress can cause tissue damage and stimulate fibrosis (Bataller and Brenner, 2005; Rehman et al., 2016; Unsal et al., 2021). Therefore, we measured hepatic 4-hydroxynoneal (4-HNE) adduct formation, an indicator of lipid peroxidation, after CCl_4_ treatment. After vehicle treatment, weak bands of 4-HNE adducts were present (Figure 4A). After 6 weeks of CCl_4_ treatment, the density of 4-HNE adducts bands increased 91% (Figures 4A, B). With LP340 treatment, 4-HNE did not increase (Figures 4A, B).

Transforming growth factor-beta (TGFβ) is a potent profibrotic cytokine (Bataller and Brenner, 2005; Xu et al., 2016; Dewidar et al., 2019). TGFβ1 expression in liver increased ∼300% after CCl_4_ treatment in male mice receiving vehicle but only increased ∼80% in mice receiving LP340 (Figures 4A, C). Expression of mothers against decapentaplegic homolog-2,3 (Smad2,3), the major TGFβ downstream signaling molecules (Xu et al., 2016; Dewidar et al., 2019), was not altered by CCl_4_ or LP340 treatment (Figures 4A, D). By contrast, the phospho-Smad2,3 (pSmad2,3)/Smad2,3 ratio increased ∼85% after CCl_4,_ indicating Smad2,3 activation (Figures 4A, E). LP340 blunted CCl_4_-induced Smad2,3 activation (Figure 4). In female mice, LP340 also blunted TGFβ1 expression after CCl_4_ treatment (Supplementary Material Supplementary Figure S2D, S2F).

Matrix metalloproteinases (MMPs) are proteinases that degrade the extracellular matrix, thus suppressing and reversing fibrosis (Duarte et al., 2015). In male mice, MMP9, a major MMP in the liver, was not significantly altered by CCl_4_ or LP340 (Figures 4A, F).

### 3.5 LP340 inhibited microRNA-23a but increased SnoN after CCl_4_ treatment

**FIGURE 5:**
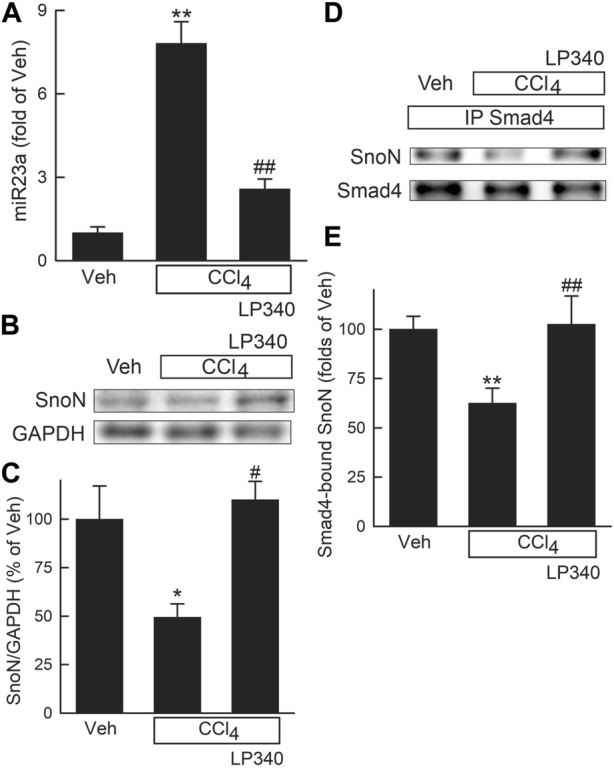
LP340 decreases microRNA-23a and increases SnoN expression and SnoN/Smad4 complex formation after CCl_4_ treatment. Male mice were treated, and livers were collected as described in Figure 1. **(A)** hepatic microRNA-23a (miR23a) detected by qPCR. **(B)** representative immunoblots of Ski-novel protein (SnoN) and housekeeping protein GAPDH. **(C)** quantification of SnoN. **(D)** representative immunoblots of Smad4-bound SnoN detected after immunoprecipitation (IP). **(E)** quantification of Smad4-bound SnoN. *, *p* < 0.05 vs. vehicle; **, *p* < 0.01 vs. vehicle; #, *p* < 0.05 vs. CCl_4_ without LP340; ##, *p* < 0.01 vs. CCl_4_ without LP340. Data are means ± SEM (n = 4/group).

Previous studies showed that downregulation of miR23a inhibits TGFβ signaling by increasing Ski-related novel protein-N (SnoN) (Xu et al., 2018). We therefore explored if inhibition of HDAC1,2,3 alters miR23a expression. In male mice, miR23a increased 6.8-fold after CCl_4_ treatment, but only increased 1.6-fold in mice with LP340 treatment (Figure 5A). SnoN is a negative regulator of TGF-β/Smad signaling (Zeglinski et al., 2015). SnoN decreased by half after CCl_4_ treatment, which LP340 reversed (Figures 5B, C). SnoN directly interacts with Smad4, thus interfering formation of the Smad4 and pSmad2/3 complex (Wu et al., 2002; Zeglinski et al., 2015). SnoN-Smad4 interaction was examined by IP of Smad4 followed by IB for SnoN. After CCl_4_ treatment, SnoN co-immunoprecipitation with Smad4 decreased ∼40%, which LP340 reversed (Figures 5D, E).

### 3.6 LP340 decreases liver injury and inflammation after bile duct ligation

**FIGURE 6:**
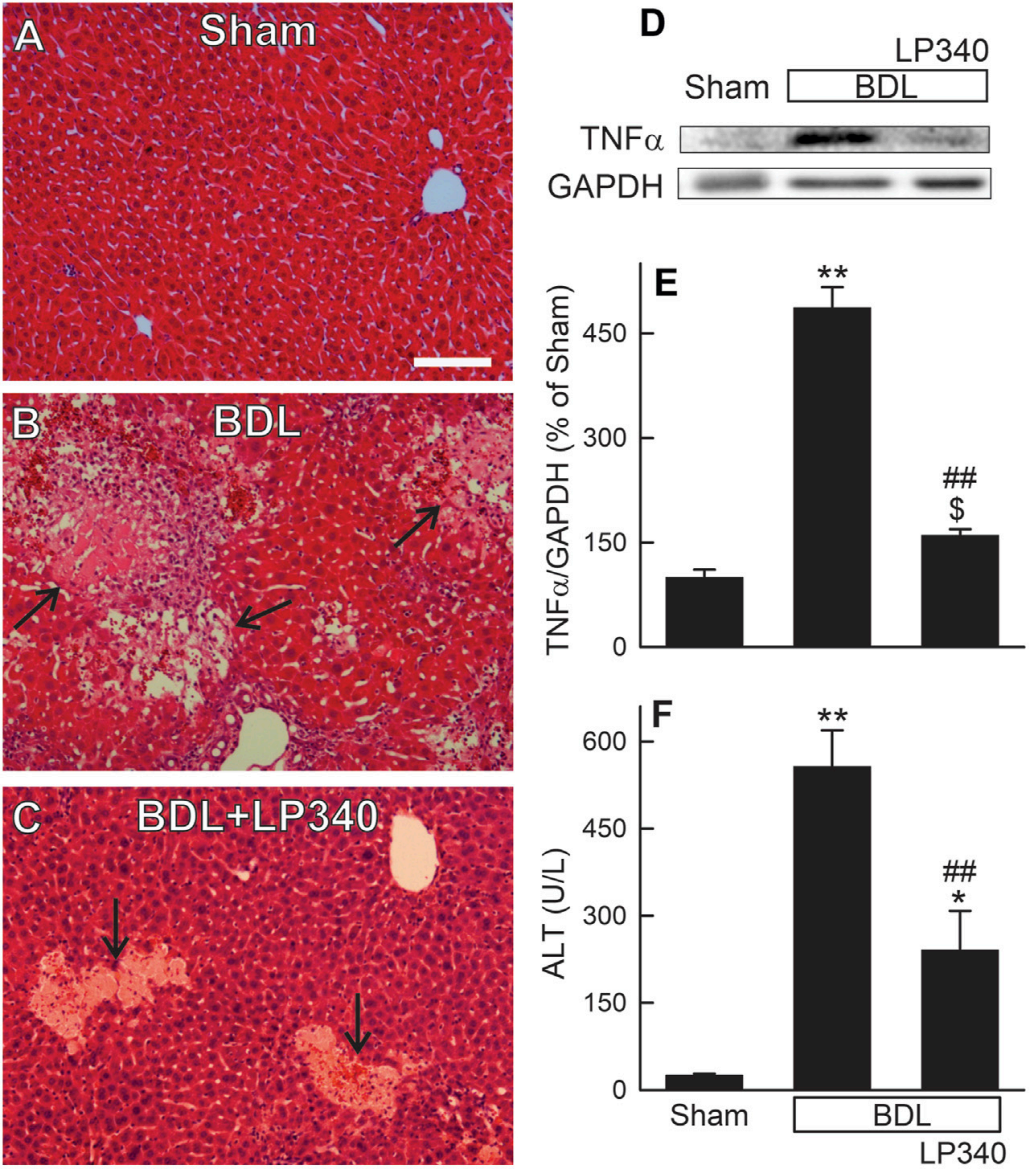
LP340 decreases liver injury and inflammation after bile duct ligation. Blood and livers were collected 2 weeks after BDL or sham operation (Sham). **(A–C)** representative images of H&E-stained liver sections. Arrows identify necro-inflammatory foci. Bar is 50 µm. **(D)** representative immunoblot images of TNFα and GAPDH; **(E)** quantification of TNFα immunoblots; **(F)** serum ALT; $, *p* = 0.052 vs. sham; *, *p* < 0.05 vs. sham; **, *p* < 0.01 vs. sham; ##, *p* < 0.01 vs. BDL without LP340. Data are means ± SEM (n = 3–4/group).

Hepatic cholestatic injury is typically associated with occurrence of portal fibrosis. Accordingly, we examined whether LP340 also inhibits BDL-induced cholestatic liver injury. In male mice, liver histology revealed normal liver architecture after sham-operation (Figure 6A). After BDL, numerous areas of focal necrosis developed (Figure 6B). Moreover, leukocyte infiltration increased markedly within and/or around focal necrosis, forming necro-inflammatory foci. In mice with LP340 treatment, focal necrosis areas were smaller and leukocyte infiltration within and/or around focal necrosis was markedly decreased (Figure 6C). Consistent with increased leukocyte infiltration in liver sections after BDL, proinflammatory cytokine TNFα increased by 387% (Figures 6D, E). With LP340 treatment, TNFα increased only by 60% (Figures 6D, E).

Serum ALT averaged 26 U/L (Figure 6F) after sham-operation. After BDL, ALT increased to ∼560 U/L at 2 weeks after BDL (Figure 6F). When mice were treated with LP340, ALT after BDL only increased to ∼240 U/L (Figure 6F). Together, these data showed that LP340 also decreased liver injury and inflammation after BDL.

### 3.7 LP340 decreases ductular reactions after bile duct ligation

**FIGURE 7:**
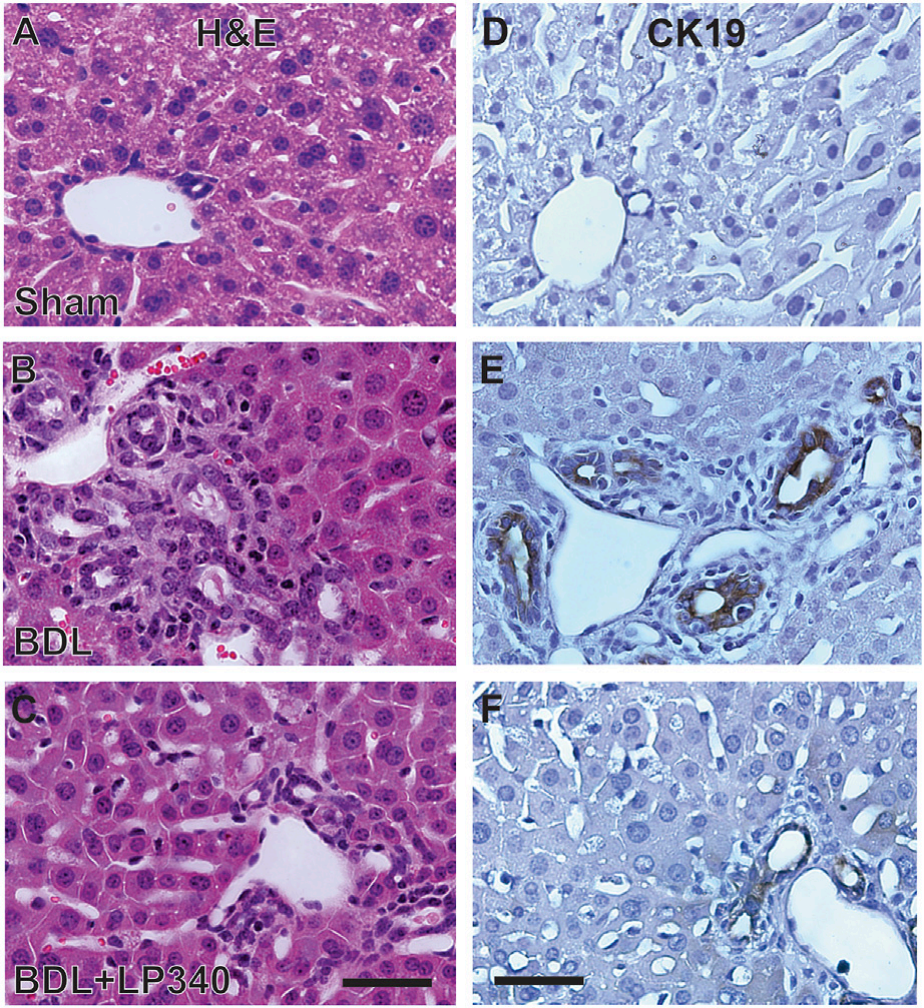
LP340 decreases ductular reactions after bile duct ligation. Livers were collected 2 weeks after BDL or sham operation (Sham). **(A–C)** representative images of liver histology after H&E staining. **(D–F)** representative images of immunohistological staining for CK19. Bars are 25 μm. n = 3–4/group.

Cholestasis typically causes a ductular reaction that is characterized by bile duct proliferation and hyperplasia. In H&E-stained liver sections from sham-operated mice, normal portal structure was observed (Figure 7A). After BDL, portal regions markedly enlarged with increased numbers of bile ducts as well as some bile ducts enlarging in size (Figure 7B). Connective tissue around portal tracts also increased (Figure 7B). Cholangiocyte proliferation was also detected by immunohistological staining for CK19, a marker of cholangiocytes (Figures 7D–F). After BDL, CK19-positive cells markedly increased, consistent with occurrence of ductular reactions (Figure 7B), which LP340 attenuated (Figures 7C, F).

### 3.8 LP340 decreases liver fibrosis after bile duct ligation

**FIGURE 8:**
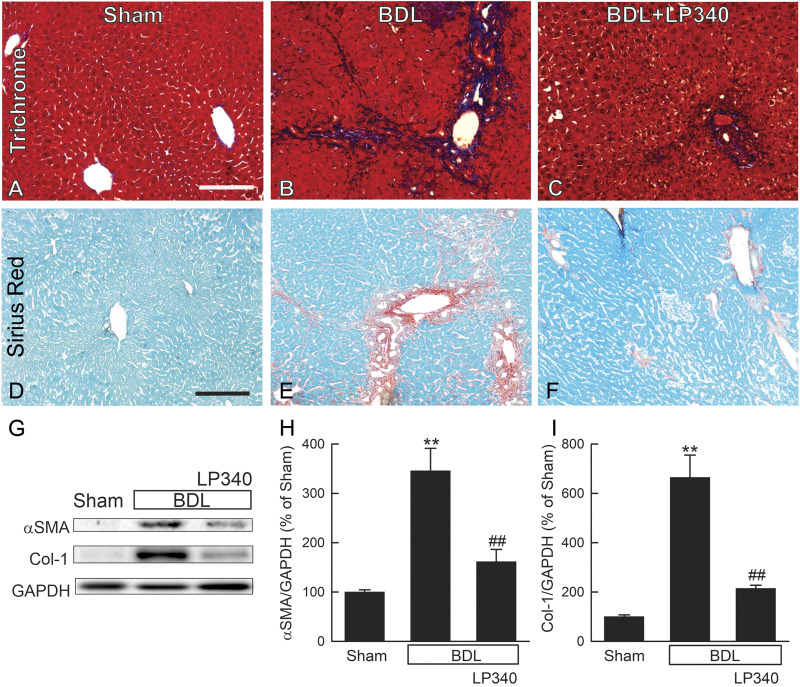
LP340 decreases liver fibrosis after bile duct ligation. Livers were collected 2 weeks after BDL or sham operation (Sham). **(A–C)** representative images of trichrome-stained liver sections. Bar is 100 µm. **(D–F)** representative images of Sirius red/Fast green-stained liver sections. Bar 100 µm. **(G)** representative immunoblots for α-smooth muscle actin (αSMA), collagen-1 (Col-1), and housekeeping protein GAPDH. **(H,I)** quantification of αSMA and Col-1 immunoblots by densitometry. **, *p* < 0.01 vs. sham; ##, *p* < 0.01 vs. BDL without LP340. Data are means ± SEM (n = 4/group).

Liver fibrosis was revealed by trichrome and Sirius-red staining in liver sections. In sham mice, trichrome and Sirius-red labeling was confined to portal tracts and around large vessels (Figures 8A, D). After BDL in male mice, trichrome and Sirius-red staining became more intense and more widely distributed in enlarged portal areas, consistent with portal fibrosis (Figures 8B, E). Bridging fibrosis also occurred in some areas. LP340 treatment attenuated these fibrotic changes after BDL (Figures 8C, F).

αSMA expression increased ∼246% after BDL, indicating HSC activation (Figures 8G, H). Collagen-1 expression increased by ∼560% after BDL, indicating fibrogenic process (Figures 8G, I). With LP340 treatment, αSMA and collagen-1 expression increased only 62% and 114%, respectively, after BDL.

### 3.9 LP340 decreases oxidative stress and microRNA-23a, increases SnoN expression, and inhibits TGFβ/Smad signaling after bile duct ligation

**FIGURE 9:**
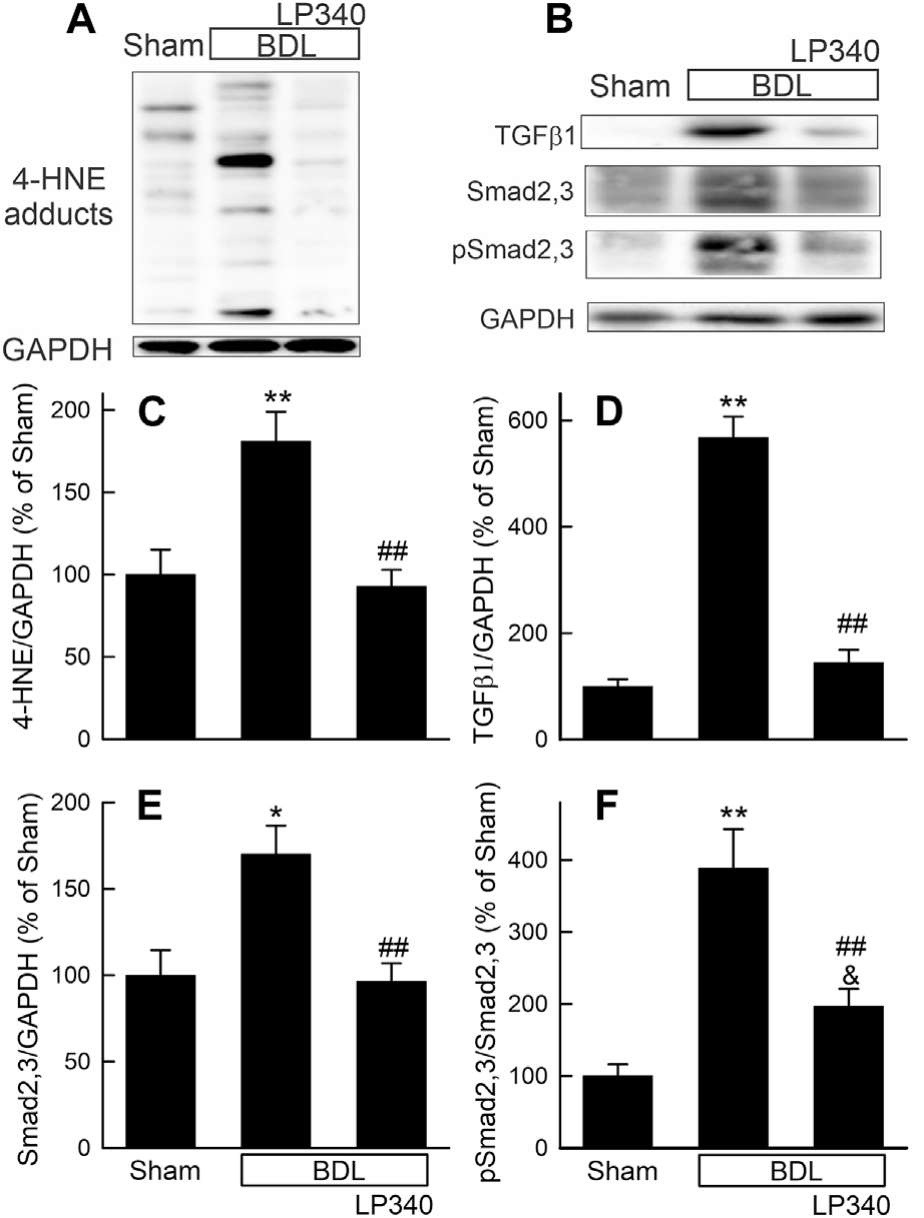
LP340 decreases oxidative stress and TGFβ/Smad signaling after bile duct ligation. Livers were collected 2 weeks after BDL or sham operation (Sham). **(A,B)** representative immunoblot for 4-HNE adducts, TGFβ1, Smad2,3, pSmad2,3 and housekeeping protein GAPDH. **(C–F)** quantification of 4-HNE adducts, TGFβ1, Smad2,3, pSmad2,3 immunoblots. *, *p* < 0.05 vs. sham; **, *p* < 0.01 vs. sham; &, *p* = 0.088 vs. sham; ##, *p* < 0.01 vs. BDL without LP340. Data are means ± SEM (n = 4/group).

Oxidative stress occurs in cholestatic liver injury and fibrosis (Zhong et al., 2002; Zhong et al., 2003; Heidari and Niknahad, 2019). We examined if LP340 decreases oxidative stress after BDL. In extracts of livers from sham-operated mice, Western blotting showed only weak bands of 4-HNE adducts (Figure 9A). After BDL, 4-HNE adducts increased 81%, which LP340 blocked (Figures 9A, C).

**FIGURE 10:**
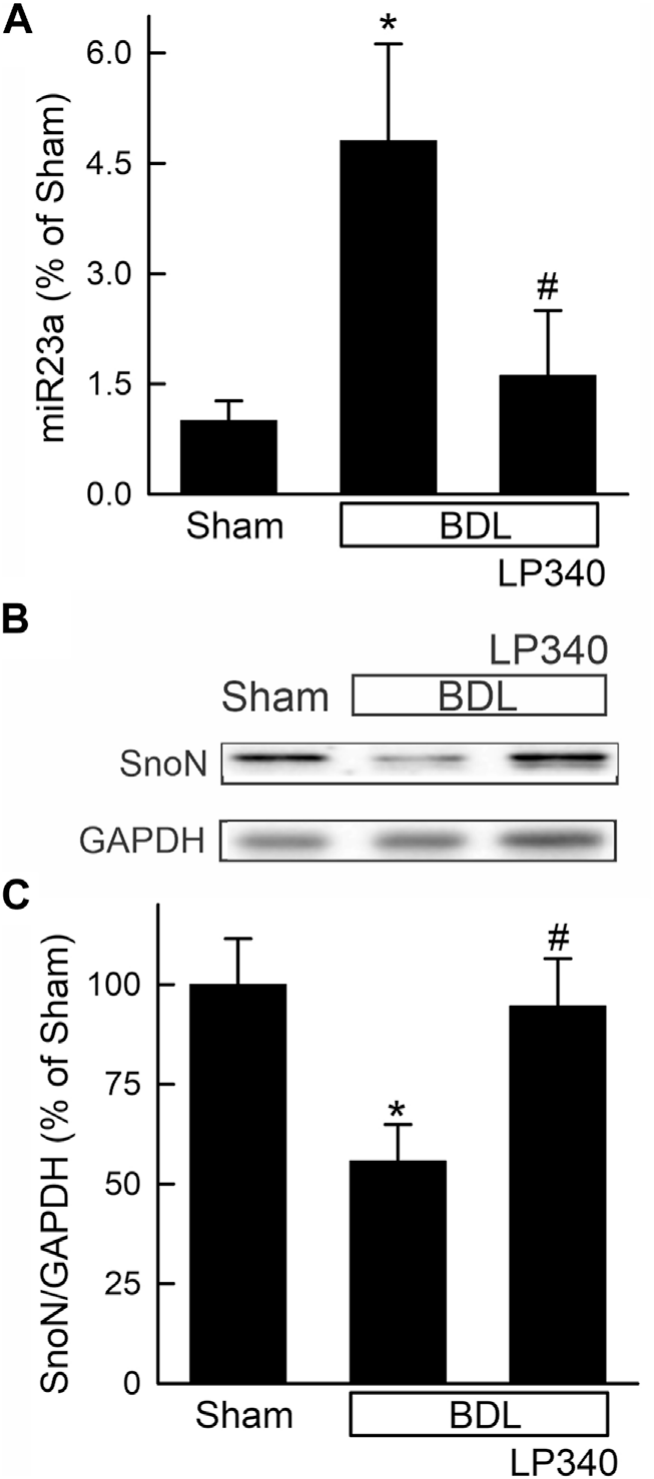
LP340 decreases microRNA-23a and increases SnoN after bile duct ligation. Livers were collected 2 weeks after BDL or sham operation (Sham). **(A)** hepatic miR23a detected by qPCR. **(B)** representative immunoblots for SnoN and housekeeping protein GAPDH. **(C)** quantification of SnoN immunoblots. *, *p* < 0.05 vs. sham; #, *p* < 0.05 vs. BDL without LP340. Data are means ± SEM (n = 4/group).

We also examined alterations in the TGFβ/Smad pathway after BDL. Hepatic TGFβ1 expression increased ∼470% after BDL compared to sham operation (Figures 9B, D), and Smad2,3 expression increased ∼70% after BDL (Figures 9B, E). Phospho-Smad2,3 (pSmad2,3) was barely detectable in sham-operated mice and increased markedly after BDL (Figures 9B, E). The ratio of pSmad2,3 to Smad2,3 increased ∼290% after BDL, indicating Smad2,3 activation (Figure 9F). LP340 blunted both TGFβ1 and Smad2,3 expression as well as Smad2,3 activation (Figures 9B–F). After BDL, miR23a increased 3.8-fold, whereas SnoN decreased ∼44% (Figures 10A–C). LP340 blunted alterations of both miR23a and SnoN (Figure 10).

### 3.10 LP340 inhibits hTERT-HSC activation *in vitro*

**FIGURE 11:**
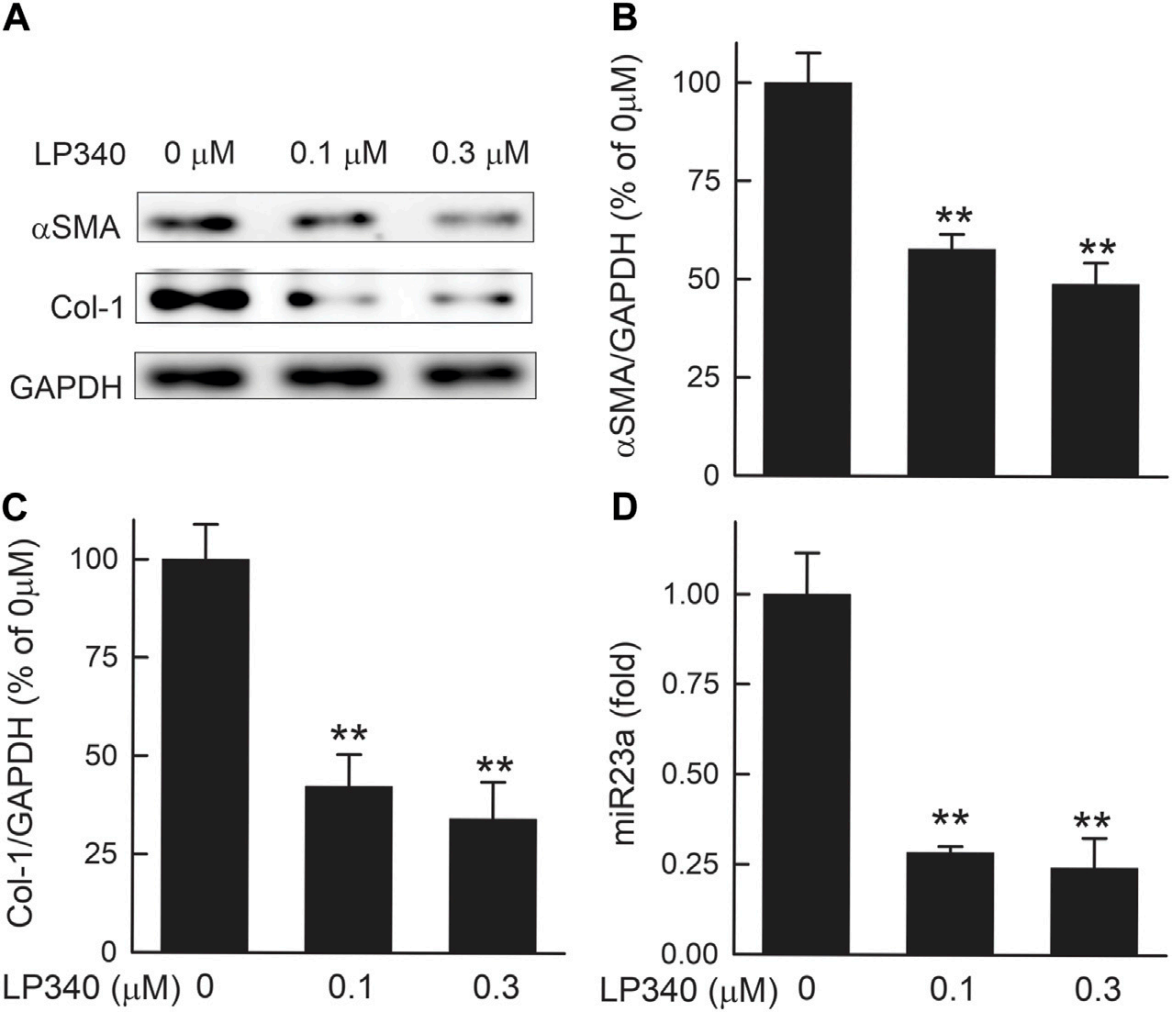
LP340 suppresses hTERT-HSC activation and miR340 formation *in vitro.* hTERT-HSC were cultured in DMEM medium with 0.5% FBS with or without LP340 (0.1 and 0.3 µM) for 48 h. **(A)** representative immunoblots for α-smooth muscle actin (αSMA), collagen-1 (Col-1), and housekeeping protein GAPDH. **(B,C)** quantification of αSMA and Col-1 immunoblots by densitometry, **(D)** miR23a detected by qPCR. **, *p* < 0.01 vs. 0 µM LP340. Data are means ± SEM (n = 3/group).

HSC undergo spontaneous activation during culture. In the lysates of hTERT-HSC after 48 h culture without LP340, overt expression of αSMA and collagen-1 occurred (Figures 11A–C), indicating activation of these cells. With exposure to 0.1 and 0.3 µM LP340, αSMA expression decreased by 42% and 51%, and collagen-1 expression decreased by 58% and 66%, indicating suppression of HSC activation in a dose-dependent faction (Figures 11A–C). LP340 at 0.1 and 0.3 µM also decreased miR23a expression in hTERT-HSC by 72% and 76%, respectively (Figure 11D). These results demonstrate that LP340 directly inhibits miR23a expression and HSC activation *in vitro*.

## 4 Discussion

### 4.1 LP340 protects against hepatic fibrosis *in vivo*

Liver fibrosis is a major medical problem with high morbidity and mortality (Friedman, 2003; Asrani et al., 2019). Importantly, insidious initiation and development of liver fibrosis substantially delay the treatment of underlying diseases. Therefore, antifibrotic treatment is much needed to decrease mortality caused by liver fibrosis (Scaglione et al., 2015). Many antifibrotic treatments targeting various fibrotic pathways have been assessed in liver fibrosis, such as angiotensin-converting enzyme inhibitors, angiotensin receptor blockers, interferon, pioglitazone, colchicine, farglitazar, simtuzumab, non-coding RNAs, pegbelfermin, natural herbs, and transplantation of stem cells (Friedman, 2015; Rockey et al., 2015; Trautwein et al., 2015). Unfortunately, only a very few of these treatments show modest effects in clinical trials, whereas most other treatments are ineffective (Friedman, 2015; Malaguarnera et al., 2015; Rockey et al., 2015; Trautwein et al., 2015; Zhang et al., 2023). The lack of broadly effective treatment for fibrosis continues to fuel the search for new molecular targets of fibrosis and effective drugs for prevention and therapy.

Recently, a new class of hydrazide-based HDACi targeting Class I HDAC was discovered by high-throughput screening, although the potencies of these initial hydrazide-based inhibitors was weaker than the first generation HDACi vorinostat (Wang et al., 2015). Through medicinal chemistry refinements, we developed novel HDACi with an additive inhibition mechanism via interaction with both competitive and allosteric sites of HDAC, resulting in higher potency and more specific target engagement (McClure et al., 2016; Li et al., 2018; Jiang et al., 2022). These new HDACi are highly potent (effective at low nanomolar levels) and have better isoform selectivity for HDAC1,2,3 and better PK properties than current FDA-approved HDACi on the market, especially in oral bioavailability and total systemic exposure. For example, the EC50s of LP340 for inhibiting HDAC1, 2, and 3 are 3.52, 18.14 and 0.38 nM respectively but are >10,000 nM for other HDACs (Jiang et al., 2022). LP340 can achieve oral bioavailability greater than 95%, and the maximum concentration in blood it can achieve (Cmax) is greater than 100 µM (Jiang et al., 2022).

In this study, we explored whether LP340 can be used as anti-fibrotic therapy in two mouse models of hepatic fibrosis–CCl_4_ and BDL, which are most widely used models for pericentral and portal fibrosis, respectively. We showed that both CCl_4_ and BDL caused necrosis, increased ALT release, proinflammatory cytokine formation, and leukocyte infiltration, indicating liver injury and inflammatory responses (Figure 1; Figure 2; Figure 6). LP340 blunted all these alterations. CCl_4_ treatment caused primarily pericentral fibrosis, whereas BDL caused mostly portal fibrosis (Figure 3; Figure 8), as expected. Bridging fibrosis occurred in each model as revealed by collagen staining with both trichrome and Sirius-red (Figure 3; Figure 8). Expression of αSMA and collagen-1 markedly increased after both CCl_4_ and BDL, demonstrating HSC activation and the fibrogenic process at molecular level (Figure 3; Figure 8). LP340 decreased fibrosis as revealed histologically and by molecular markers (Figure 3; Figure 8). Moreover, LP340 directly inhibited hTERT-HSC activation *in vitro* (Figure 11). Overall, these results suggest that LP340 not only decreases liver injury but is also a promising therapy for both pericentral and portal fibrosis. Importantly, in BDL, LP340 gavage was started right after surgery, whereas in the 6-week CCl_4_ model, LP340 was given only in the last 2 weeks, suggesting LP340 is effective both as a prevention and treatment strategy.

### 4.2 LP340 decreases oxidative stress

Liver fibrosis results from wound-healing responses to repeated injury during the progression of most chronic liver diseases (Bataller and Brenner, 2005; Zhang et al., 2023). Many cellular and molecular signals contribute to the initiation and progression of liver fibrosis, including cell death, oxidative stress, mitochondrial dysfunction, inflammatory processes, proinflammatory/profibrotic cytokines, vasoactive substances, adipokines, microRNAs, ductular reactions, and genetic factors (Gabele et al., 2003; Bataller and Brenner, 2005; Zhang et al., 2023). Chronic liver injury stimulates a multicellular response involving many hepatic resident and infiltrating cells, which release a variety of mediators to stimulate inflammatory, proliferative, and profibrogenic responses (Guo et al., 2009; Brenner et al., 2012; Higashi et al., 2017). In response to these mediators, HSC become activated and transform into myofibroblast-like cells that acquire proinflammatory, contractile, and fibrogenic properties. Activated HSCs migrate to and accumulate in sites where tissue injury occurs, producing a collagen-rich ECM to repair damaged liver tissue (Brenner et al., 2012; Higashi et al., 2017). However, dysregulated, excessive profibrogenic processes eventually lead to fibrosis/cirrhosis. By contrast, removal of activated HSCs by apoptosis and increased collagenolytic activity by upregulation of MMPs lead to resolution of fibrosis (Arthur, 2002; Bataller and Brenner, 2005).

The liver is an important site of ROS production due to its active metabolic and detoxification activities (Allameh et al., 2023). Oxidative stress develops in prevalent infectious, metabolic, drug-induced, cholestatic, autoimmune, and genetic liver diseases, such as viral hepatitis B or C, alcohol-associated liver disease, metabolic dysfunction-related steatotic liver disease, primary biliary cirrhosis, and Wilson’s disease (Allameh et al., 2023). ROS attack many biologically important macromolecules (e.g., lipids, proteins, DNA) and cause mitochondrial dysfunction, leading to cell injury and death (Hensley et al., 2000; Kim et al., 2003; Corpas and Barroso, 2013). Experimental and clinical evidence shows that oxidative stress also plays a critical role in the development of fibrosis by stimulating/amplifying inflammatory and profibrotic responses via increased expression and activation of proinflammatory/profibrotic mediators (e.g., TNFα, TGFβ, IL-1β) (Sanchez-Valle et al., 2012; Allameh et al., 2023). Oxidative stress also causes senescence in cholangiocytes and stimulates senescence-related bile ductular reactions, thus causing release of cholangiokines (e.g., TGFβ, connective tissue growth factor) that stimulate fibrosis in cholestatic liver disease (Nakanuma et al., 2015; Carpino et al., 2017; Sato et al., 2019; Cai et al., 2023). ROS and products of lipid peroxidation stimulate quiescent HSC to transdifferentiate into an activated, highly proliferative myofibroblast-like phenotype (Svegliati-Baroni et al., 2001a; Svegliati-Baroni et al., 2001b; Gandhi, 2012). Antioxidant plant polyphenols, over-expression of mitochondrial superoxide dismutase-2 (SOD2, which degrades superoxide), mitochondrial targeting antioxidant MitoQ, and activation of aldehyde dehydrogenase-2 (ALDH2, metabolically detoxifies lipid peroxidation-produced toxic aldehydes) all attenuated liver injury and fibrosis after BDL or CCl_4_ treatment (Zhong et al., 2002; Zhong et al., 2003; Wimborne et al., 2019).

**FIGURE 12:**
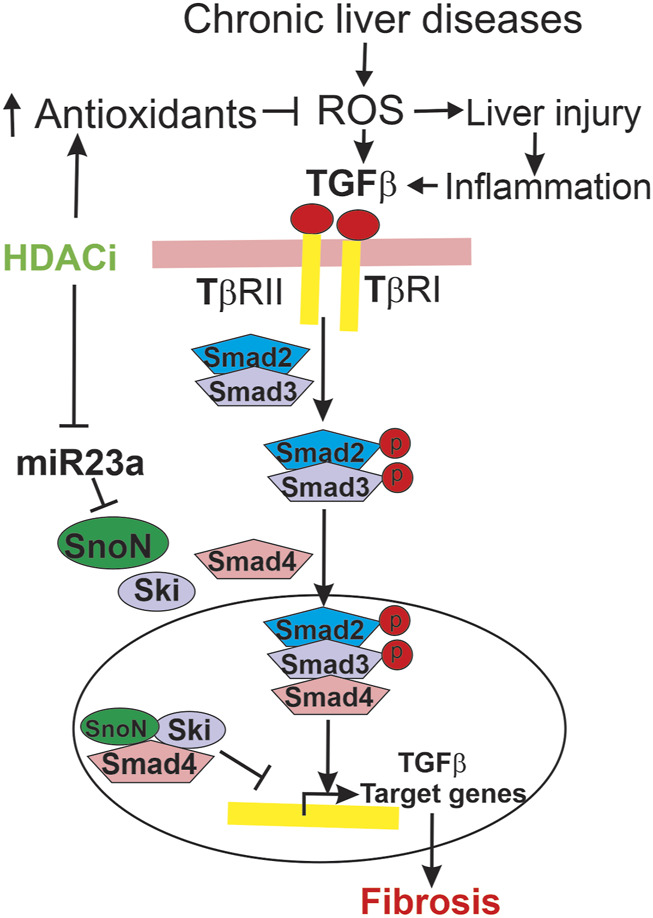
Mechanisms by which LP340 ameliorates liver fibrosis. Chronic liver diseases increase ROS formation, which causes cell injury and death, stimulate inflammatory responses, and increase TGFβ. Inflammatory responses also stimulate TGFβ formation. TGFβ binds to its receptors, causing phosphorylation of Smad2 and Smad3. Phosphorylated Smad2 and Smad3 form a heterocomplex with Smad4, and this complex translocates to the nucleus to upregulate transcription of TGFβ target genes, which causes HSC activation and fibrosis. SnoN and Ski, endogenous inhibitors of TGFβ/Smad signaling, compete for Smad4 with pSmad2/3 thus inhibiting pSmad2/3 nuclear translocation and TGFβ target gene transcription. Alternatively or additionally, SnoN and Ski form an inhibitory complex with Smad4 that binds to the promoter of TGFβ target genes, thus inhibiting TGFβ target gene transcription. miR23a inhibits SnoN expression. LP340 decreases oxidative stress, most likely by increasing antioxidant protein formation. Inhibition of oxidative stress would decrease liver injury and subsequent proinflammatory and profibrotic responses. Moreover, LP340 suppresses miR23a formation, which increases SnoN and its binding to Smad4, thus inhibiting TGFβ/Smad signaling-induced HSC activation and liver fibrosis.

In this study, oxidative stress occurred after CCl_4_ treatment and BDL, as indicated by increased 4-HNE adduct formation (Figure 4; Figure 9). LP340 blunted increases of 4-HNE, suggesting that LP340 decreases oxidative stress (Figure 12). Previous studies showed that HDACs epigenetically regulate the kelch-like ECH associated protein 1(Keap1)/nuclear factor E2-related factor 2 (Nrf2)/antioxidant pathway (Guo et al., 2015). In cultured Raw-264.7 macrophages, HDAC inhibition decreases Keap1 expression, a suppressor of Nrf2 (Wang et al., 2012). Under normal conditions, Keap1 and Nrf2 form a complex that remains localized in the cytosol. When Keap1 decreases, Keap1/Nrf2 dissociation leads to Nrf2 translocation into nuclei and then increased expression of many antioxidant proteins that contain antioxidant response elements (AREs) in their promoter regions (Kensler et al., 2007; Wang et al., 2012). TSA, a pan HDAC inhibitor, decreases infarct volume after stroke in wild-type mice, and this effect is abolished by Nrf2-deficiency (Wang et al., 2012). Consistent with an antioxidant effect of HDACi, we recently showed that LP342 decreases 4-HNE formation, increases expression of antioxidant proteins, and protects against hepatic ischemia/reperfusion injury (Samuvel et al., 2023). Therefore, LP340 likely inhibits liver injury and fibrosis, at least in part, by increasing expression of antioxidant proteins through activation of the Nrf2 pathway, thus inhibiting oxidative stress.

### 4.3 LP340 inhibits liver fibrosis by altering microRNA-23a, SnoN, and TGFβ/Smad signaling

In addition to attenuation of liver injury and subsequent inflammatory responses, LP340 may inhibit fibrosis by epigenetically decreasing miR23a expression thus increasing SnoN, a potent suppressor of TGFβ signaling (Figure 12). TGFβ is a master profibrogenic cytokine that stimulates HSC activation, proliferation, fibrogenesis, and migration (Dewidar et al., 2019). TGFβ is produced not only by macrophages and cholangiocytes but also by activated HSC, thus acting as an autocrine/paracrine factor. Binding of TGFβ to its receptor (TβR) leads to phosphorylation of Smad2 and Smad3 (R-Smads). Phosphorylated Smad2 and Smad3 form a heterocomplex with Smad4 (co-Smad). This complex translocates to the nucleus and recruits additional transcriptional coactivators to promote transcription of TGFβ target genes, such as the gene for αSMA (Figure 12) (Derynck and Budi, 2019; Dewidar et al., 2019). HDACs enhance TGFβ-induced trans-differentiation of HSC to myofibroblasts and ECM production (Chen et al., 2015). During HSC trans-differentiation, the global acetylation of both histone H3 and H4 progressively decreases, suggesting increased HDAC activity (Qin and Han, 2010). In this study, we showed that selective and potent HDAC1,2,3 inhibitor LP340 suppresses TGFβ/Smad signaling and HSC activation *in vivo* (Figure 4; Figure 9).

LP340 possibly works by increasing formation of SnoN, a protein that interacts with Ski as transcriptional corepressors of Smad proteins, thus suppressing TGFβ/Smad signaling (Deheuninck and Luo, 2009; Zeglinski et al., 2015; Tecalco-Cruz et al., 2018). Ski and SnoN interact simultaneously with Smad4 (Wu et al., 2002; Deheuninck and Luo, 2009; Zeglinski et al., 2015). Binding of Ski/SnoN to Smad4 results in displacement of phospho-Smad2,3 from Smad4, thus disrupting nuclear translocation of Smad heteromeric complexes and subsequent TGFβ target gene transcription (Figure 12) (Wu et al., 2002). Another model proposes that Ski and SnoN block TGFβ signaling by forming an inhibitory complex with Smad4 that binds to the promoters of TGFβ target genes and recruits additional corepressors to inhibit TGFβ target gene expression (Figure 12) (Tecalco-Cruz et al., 2018). Other studies show that Ski and SnoN proteins also directly interact with TβRI, thus inhibiting pSmad2,3-Smad4 complex formation (Ferrand et al., 2010). Many studies have shown inhibitory effects of Ski/SnoN on fibrosis in various organs (Zeglinski et al., 2015). For example, SnoN upregulation inhibits TGFβ/Smad signaling and ameliorates renal fibrosis in diabetic rats (Liu et al., 2017). Docosahexaenoic acid, an essential n-3 polyunsaturated fatty acid, increases SnoN, thus decreasing paraquat-induced pulmonary fibrosis (Chen et al., 2013). Cpd861, a herbal compound that increases SnoN, alleviates liver fibrosis (Cai et al., 2006; Chi et al., 2018). Consistently, in this study we observed that SnoN expression markedly decreased after BDL and CCl_4_ treatment in association with decreased formation of SnoN/Smad4 complexes, increased TGFβ/Smad signaling, and liver fibrosis, whereas LP340 increased SnoN and inhibited hepatic fibrosis after BDL and CCl_4_ treatment (Figure 5; Figure 10).

LP340 increases SnoN expression likely through decreasing miR23a. miR23a plays an important role in initiation and progression of tumors (Wang et al., 2018). miR23a increases in liver fibrosis and hepatocellular carcinoma (Bao et al., 2014; Dong et al., 2019). In this study, we showed that miR23a increases after BDL and CCl_4_ treatment as well as in activated human HSC cell line in culture (Figure 5; Figure 10; Figure 11), which is consistent with previous reports (Dong et al., 2019). Interestingly, a previous study shows that miR23a increases TGFβ signaling by decreasing transcription of SnoN (Xu et al., 2018). Therefore, we examined the effects of inhibition of HDAC1,2,3 by LP340 on miR23a. We showed that LP340 inhibited the increase of miR23a and reversed decreases of SnoN and formation of the SnoN/Smad4 complexes after BDL and CCl_4_ and in cultured human HSC cell line (Figure 5; Figure 10; Figure 11).

Taken together, our findings show that LP340, the lead of a new generation of selective HDACi for HDAC1, 2 and 3, alleviates liver fibrosis either preventively or when given as therapy. LP340 inhibits Class I but not Class II HDACs *in vivo*. These novel HDACi not only protect against liver injury but also directly inhibit HSC activation. HDACi likely protect against liver injury and fibrosis by epigenetic upregulation of antioxidant proteins. Additionally, HDACi inhibit fibrosis by suppression of miR23a expression, thus increasing SnoN and inhibiting TGFβ signaling (Figure 12). This new generation of HDACi may represent a promising new therapy for fibrosis.

## Figures and Tables

**Table:** 

**acH3**	acetylated histone-3
**acTub**	acetylated tubulin
**ALT**	alanine aminotransferase
**AREs**	antioxidant response elements
**αSMA**	α-smooth muscle actin
**BDL**	bile duct ligation
**CCl4**	Carbon tetrachloride
**CK19**	cytokeratin
**ECM**	extracellular matrix
**FBS**	fetal bovine serum
**GAPDH**	glyceraldehyde-3-phosphate dehydrogenase
**HDAC**	histone deacetylase
**HDACi**	histone deacetylase inhibitor
**hTERT-HSC**	immortal human hepatic stellate cells
**H&E**	hematoxylin-eosin
**4-HNE**	4-hydroxynonenal
**HSC**	hepatic stellate cell
**Keap1**	kelch-like ECH associated protein 1
**miR23a**	microRNA-23a
**MMP9**	matrix metalloproteinase-9
**MPO**	myeloperoxidase
**NFκB**	nuclear factor-κB
**Nrf2**	nuclear factor E2-related factor 2
**p53**	tumor protein P53
**PK**	pharmacokinetics
**ROS**	reactive oxygen species
**SAHA**	suberoylanilide hydroxamic acid
**Ski**	Sloan-Kettering Institute
**Smad**	mothers against decapentaplegic homolog
**SnoN**	Ski-related novel protein-N
**TGFβ**	transforming growth factor-β
**TNFα**	tumor necrosis factor-α
**TSA**	trichostatin A

## Data Availability

The raw data supporting the conclusion of this article will be made available by the authors, without undue reservation.

## References

[B1] Allameh A. Niayesh-Mehr R. Aliarab A. Sebastiani G. Pantopoulos K. (2023). Oxidative stress in liver pathophysiology and disease. Antioxidants (Basel) 12 (9), 1653. 10.3390/antiox12091653 37759956 PMC10525124

[B2] Arthur M. J. (2002). Reversibility of liver fibrosis and cirrhosis following treatment for hepatitis C. Gastroenterology 122 (5), 1525–1528. 10.1053/gast.2002.33367 11984538

[B3] Asrani S. K. Devarbhavi H. Eaton J. Kamath P. S. (2019). Burden of liver diseases in the world. J. Hepatol. 70 (1), 151–171. 10.1016/j.jhep.2018.09.014 30266282

[B4] Bao L. Zhao J. Dai X. Wang Y. Ma R. Su Y. (2014). Correlation between miR-23a and onset of hepatocellular carcinoma. Clin. Res. Hepatol. Gastroenterol. 38 (3), 318–330. 10.1016/j.clinre.2013.12.002 24417970

[B5] Bataller R. Brenner D. A. (2005). Liver fibrosis. J. Clin. Invest. 115 (2), 209–218. 10.1172/JCI24282 15690074 PMC546435

[B6] Brenner D. A. Kisseleva T. Scholten D. Paik Y. H. Iwaisako K. Inokuchi S. (2012). Origin of myofibroblasts in liver fibrosis. Fibrogenes. Tissue Repair 5 (Suppl. 1), S17. 10.1186/1755-1536-5-S1-S17 PMC3368775 23259769

[B7] Cai X. Tacke F. Guillot A. Liu H. (2023). Cholangiokines: undervalued modulators in the hepatic microenvironment. Front. Immunol. 14, 1192840. 10.3389/fimmu.2023.1192840 37261338 PMC10229055

[B8] Cai Y. Shen X. Z. Zhou C. H. Wang J. Y. (2006). Abnormal expression of Smurf2 during the process of rat liver fibrosis. Chin. J. Dig. Dis. 7 (4), 237–245. 10.1111/j.1443-9573.2006.00275.x 17054587

[B9] Carpino G. Pastori D. Baratta F. Overi D. Labbadia G. Polimeni L. (2017). PNPLA3 variant and portal/periportal histological pattern in patients with biopsy-proven non-alcoholic fatty liver disease: a possible role for oxidative stress. Sci. Rep. 7 (1), 15756. 10.1038/s41598-017-15943-z 29150621 PMC5693899

[B10] Chen J. Zeng T. Zhao X. Xiea K. Bi Y. Zhong Z. (2013). Docosahexaenoic acid (DHA) ameliorates paraquat-induced pulmonary fibrosis in rats possibly through up-regulation of Smad 7 and SnoN. Food Chem. Toxicol. 57, 330–337. 10.1016/j.fct.2013.03.045 23590892

[B11] Chen P. J. Huang C. Meng X. M. Li J. (2015). Epigenetic modifications by histone deacetylases: biological implications and therapeutic potential in liver fibrosis. Biochimie 116, 61–69. 10.1016/j.biochi.2015.06.016 26116886

[B12] Chi C. Liu X. Y. Hou F. Yu X. Z. Li C. Y. Cui L. J. (2018). Herbal compound 861 prevents hepatic fibrosis by inhibiting the TGF-β1/Smad/SnoN pathway in bile duct-ligated rats. BMC Complement. Altern. Med. 18 (1), 52. 10.1186/s12906-018-2119-7 29402324 PMC5800072

[B13] Corpas F. J. Barroso J. B. (2013). Nitro-oxidative stress vs oxidative or nitrosative stress in higher plants. New Phytol. 199 (3), 633–635. 10.1111/nph.12380 23763656

[B14] Crawford J. M. (2002). “Liver cirrhosis,” in Pathology of the liver. 4 ed. (London, England: Churchill Livingstone), 575–619.

[B15] Deheuninck J. Luo K. (2009). Ski and SnoN, potent negative regulators of TGF-beta signaling. Cell Res. 19 (1), 47–57. 10.1038/cr.2008.324 19114989 PMC3103856

[B16] Derynck R. Budi E. H. (2019). Specificity, versatility, and control of TGF-β family signaling. Sci. Signal 12 (570), eaav5183. 10.1126/scisignal.aav5183 30808818 PMC6800142

[B17] Dewidar B. Meyer C. Dooley S. Meindl-Beinker A. N. (2019). TGF-Β in hepatic stellate cell activation and liver fibrogenesis-updated 2019. Cells 8 (11), 1419. 10.3390/cells8111419 31718044 PMC6912224

[B18] Dong Z. Li S. Wang X. Si L. Ma R. Bao L. (2019). lncRNA GAS5 restrains CCl(4)-induced hepatic fibrosis by targeting miR-23a through the PTEN/PI3K/Akt signaling pathway. Am. J. Physiol. Gastrointest. Liver Physiol. 316 (4), G539–G50. 10.1152/ajpgi.00249.2018 30735452

[B19] Duarte S. Baber J. Fujii T. Coito A. J. (2015). Matrix metalloproteinases in liver injury, repair and fibrosis. Matrix Biol. 44-46, 147–156. 10.1016/j.matbio.2015.01.004 25599939 PMC4495728

[B20] Ferrand N. Atfi A. Prunier C. (2010). The oncoprotein c-ski functions as a direct antagonist of the transforming growth factor-{beta} type I receptor. Cancer Res. 70 (21), 8457–8466. 10.1158/0008-5472.CAN-09-4088 20959473

[B21] Friedman S. L. (2003). Liver fibrosis -- from bench to bedside. J. Hepatol. 38 (Suppl. 1), S38–S53. 10.1016/s0168-8278(02)00429-4 12591185

[B22] Friedman S. L. (2015). Hepatic fibrosis: emerging therapies. Dig. Dis. 33 (4), 504–507. 10.1159/000374098 26159266

[B23] Gabele E. Brenner D. A. Rippe R. A. (2003). Liver fibrosis: signals leading to the amplification of the fibrogenic hepatic stellate cell. Front. Biosci. 8, d69–d77. 10.2741/887 12456323

[B24] Gandhi C. R. (2012). Oxidative stress and hepatic stellate cells: a paradoxical relationship. Trends Cell Mol. Biol. 7, 1–10. 27721591 PMC5051570

[B25] Glozak M. A. Sengupta N. Zhang X. Seto E. (2005). Acetylation and deacetylation of non-histone proteins. Gene 363, 15–23. 10.1016/j.gene.2005.09.010 16289629

[B26] Guo C. J. Pan Q. Cheng T. Jiang B. Chen G. Y. Li D. G. (2009). Changes in microRNAs associated with hepatic stellate cell activation status identify signaling pathways. FEBS J. 276 (18), 5163–5176. 10.1111/j.1742-4658.2009.07213.x 19674103

[B27] Guo Y. Yu S. Zhang C. Kong A. N. (2015). Epigenetic regulation of Keap1-Nrf2 signaling. Free Radic. Biol. Med. 88 (Pt B), 337–349. 10.1016/j.freeradbiomed.2015.06.013 26117320 PMC4955581

[B28] Hadziyannis S. J. Tassopoulos N. C. Heathcote E. J. Chang T. T. Kitis G. Rizzetto M. (2003). Adefovir dipivoxil for the treatment of hepatitis B e antigen-negative chronic hepatitis B. N. Engl. J. Med. 348 (9), 800–807. 10.1056/NEJMoa021812 12606734

[B29] Hammond C. M. Stromme C. B. Huang H. Patel D. J. Groth A. (2017). Histone chaperone networks shaping chromatin function. Nat. Rev. Mol. Cell Biol. 18 (3), 141–158. 10.1038/nrm.2016.159 28053344 PMC5319910

[B30] Heidari R. Niknahad H. (2019). The role and study of mitochondrial impairment and oxidative stress in cholestasis. Methods Mol. Biol. 1981, 117–132. 10.1007/978-1-4939-9420-5_8 31016651

[B31] Hensley K. Robinson K. A. Gabbita S. P. Salsman S. Floyd R. A. (2000). Reactive oxygen species, cell signaling, and cell injury. Free Radic. Biol. Med. 28, 1456–1462. 10.1016/s0891-5849(00)00252-5 10927169

[B32] Higashi T. Friedman S. L. Hoshida Y. (2017). Hepatic stellate cells as key target in liver fibrosis. Adv. Drug Deliv. Rev. 121, 27–42. 10.1016/j.addr.2017.05.007 28506744 PMC5682243

[B33] Jiang Y. Xu J. Yue K. Huang C. Qin M. Chi D. (2022). Potent hydrazide-based HDAC inhibitors with a superior pharmacokinetic profile for efficient treatment of acute myeloid leukemia *in vivo* . J. Med. Chem. 65 (1), 285–302. 10.1021/acs.jmedchem.1c01472 34942071

[B34] Kensler T. W. Wakabayashi N. Biswal S. (2007). Cell survival responses to environmental stresses via the Keap1-Nrf2-ARE pathway. Annu. Rev. Pharmacol. Toxicol. 47, 89–116. 10.1146/annurev.pharmtox.46.120604.141046 16968214

[B35] Kiernan J. A. (2015) Histological and histochemical methods. 5th Edition. Banbury, UK: Scion.

[B36] Kiernan R. Bres V. Ng R. W. Coudart M. P. El M. S. Sardet C. (2003). Post-activation turn-off of NF-kappa B-dependent transcription is regulated by acetylation of p65. J. Biol. Chem. 278 (4), 2758–2766. 10.1074/jbc.M209572200 12419806

[B37] Kim J. S. He L. Qian T. Lemasters J. J. (2003). Role of the mitochondrial permeability transition in apoptotic and necrotic death after ischemia/reperfusion injury to hepatocytes. Curr. Mol. Med. 3 (6), 527–535. 10.2174/1566524033479564 14527084

[B38] Krishnasamy Y. Ramshesh V. K. Gooz M. Schnellmann R. G. Lemasters J. J. Zhong Z. (2016). Ethanol and high cholesterol diet causes severe steatohepatitis and early liver fibrosis in mice. PLoS One 11 (9), e0163342. 10.1371/journal.pone.0163342 27676640 PMC5038945

[B39] Kuo M. H. Allis C. D. (1998). Roles of histone acetyltransferases and deacetylases in gene regulation. Bioessays. 20 (8), 615–626. 10.1002/(SICI)1521-1878(199808)20:8<615::AID-BIES4>3.0.CO;2-H 9780836

[B40] Li G. Tian Y. Zhu W. G. (2020). The roles of histone deacetylases and their inhibitors in cancer therapy. Front. Cell Dev. Biol. 8, 576946. 10.3389/fcell.2020.576946 33117804 PMC7552186

[B41] Li X. Peterson Y. K. Inks E. S. Himes R. A. Li J. Zhang Y. (2018). Class I HDAC inhibitors display different antitumor mechanism in leukemia and prostatic cancer cells depending on their p53 status. J. Med. Chem. 61 (6), 2589–2603. 10.1021/acs.jmedchem.8b00136 29499113 PMC5908721

[B42] Liu L. Shi M. Wang Y. Zhang C. Su B. Xiao Y. (2017). SnoN upregulation ameliorates renal fibrosis in diabetic nephropathy. PLoS One 12 (3), e0174471. 10.1371/journal.pone.0174471 28350874 PMC5370123

[B43] Liu Q. Rehman H. Krishnasamy Y. Schnellmann R. G. Lemasters J. J. Zhong Z. (2015). Improvement of liver injury and survival by JNK2 and iNOS deficiency in liver transplants from cardiac death mice. J. Hepatol. 63 (1), 68–74. 10.1016/j.jhep.2015.02.017 25703084 PMC4475508

[B44] Liu Y. Wang Z. Wang J. Lam W. Kwong S. Li F. (2013). A histone deacetylase inhibitor, largazole, decreases liver fibrosis and angiogenesis by inhibiting transforming growth factor-β and vascular endothelial growth factor signalling. Liver Int. 33 (4), 504–515. 10.1111/liv.12034 23279742

[B45] Malaguarnera M. Motta M. Vacante M. Malaguarnera G. Caraci F. Nunnari G. (2015). Silybin-vitamin E-phospholipids complex reduces liver fibrosis in patients with chronic hepatitis C treated with pegylated interferon α and ribavirin. Am. J. Transl. Res. 7 (11), 2510–2518. 26807195 PMC4697727

[B46] Mannaerts I. Eysackers N. Onyema O. O. Van Beneden K. Valente S. Mai A. (2013). Class II HDAC inhibition hampers hepatic stellate cell activation by induction of microRNA-29. PLoS One 8 (1), e55786. 10.1371/journal.pone.0055786 23383282 PMC3561334

[B47] Mannaerts I. Nuytten N. R. Rogiers V. Vanderkerken K. van Grunsven L. A. Geerts A. (2010). Chronic administration of valproic acid inhibits activation of mouse hepatic stellate cells *in vitro* and *in vivo* . Hepatology 51 (2), 603–614. 10.1002/hep.23334 19957378

[B48] McClure J. J. Zhang C. Inks E. S. Peterson Y. K. Li J. Chou C. J. (2016). Development of allosteric hydrazide-containing class I histone deacetylase inhibitors for use in acute myeloid leukemia. J. Med. Chem. 59 (21), 9942–9959. 10.1021/acs.jmedchem.6b01385 27754681 PMC5260863

[B49] Meirelles Junior R. F. Salvalaggio P. Rezende M. B. Evangelista A. S. Guardia B. D. Matielo C. E. (2015). Liver transplantation: history, outcomes and perspectives. Einstein (Sao Paulo) 13 (1), 149–152. 10.1590/S1679-45082015RW3164 25993082 PMC4977591

[B50] Moran-Salvador E. Mann J. (2017). Epigenetics and liver fibrosis. Cell Mol. Gastroenterol. Hepatol. 4 (1), 125–134. 10.1016/j.jcmgh.2017.04.007 28593184 PMC5453904

[B51] Nakanuma Y. Sasaki M. Harada K. (2015). Autophagy and senescence in fibrosing cholangiopathies. J. Hepatol. 62 (4), 934–945. 10.1016/j.jhep.2014.11.027 25435435

[B52] Pinzani M. (1999). Liver fibrosis. Springer Semin. Immunopathol. 21 (4), 475–490. 10.1007/s002810000037 10945037

[B53] Powell L. W. Kerr J. F. (1970). Reversal of "cirrhosis" in idiopathic haemochromatosis following long-term intensive venesection therapy. Australas. Ann. Med. 19 (1), 54–57. 10.1111/imj.1970.19.1.54 5505522

[B54] Poynard T. McHutchison J. Manns M. Trepo C. Lindsay K. Goodman Z. (2002). Impact of pegylated interferon alfa-2b and ribavirin on liver fibrosis in patients with chronic hepatitis C. Gastroenterology 122 (5), 1303–1313. 10.1053/gast.2002.33023 11984517

[B55] Qin L. Han Y. P. (2010). Epigenetic repression of matrix metalloproteinases in myofibroblastic hepatic stellate cells through histone deacetylases 4: implication in tissue fibrosis. Am. J. Pathol. 177 (4), 1915–1928. 10.2353/ajpath.2010.100011 20847282 PMC2947286

[B56] Rahimi R. S. Rockey D. C. (2013). End-stage liver disease complications. Curr. Opin. Gastroenterol. 29 (3), 257–263. 10.1097/MOG.0b013e32835f43b0 23429468

[B57] Ramzan F. Vickers M. H. Mithen R. F. (2021). Epigenetics, microRNA and metabolic syndrome: a comprehensive review. Int. J. Mol. Sci. 22 (9), 5047. 10.3390/ijms22095047 34068765 PMC8126218

[B58] Rehman H. Liu Q. Krishnasamy Y. Shi Z. Ramshesh V. K. Haque K. (2016). The mitochondria-targeted antioxidant MitoQ attenuates liver fibrosis in mice. Int. J. Physiol. Pathophysiol. Pharmacol. 8 (1), 14–27. 27186319 PMC4859875

[B59] Rehman H. Ramshesh V. K. Theruvath T. P. Kim I. Currin R. T. Giri S. (2008). NIM811 (N-methyl-4-isoleucine cyclosporine), a mitochondrial permeability transition inhibitor, attenuates cholestatic liver injury but not fibrosis in mice. J. Pharmacol. Exp. Ther. 327, 699–706. 10.1124/jpet.108.143578 18801946 PMC2582973

[B60] Rockey D. C. Bell P. D. Hill J. A. (2015). Fibrosis--a common pathway to organ injury and failure. N. Engl. J. Med. 372 (12), 1138–1149. 10.1056/NEJMra1300575 25785971

[B61] Samuvel D. J. Krishnasamy Y. Li L. Lemasters J. J. Chou C. J. Zhong Z. (2023). LP342, a novel histone deacetylase inhibitor, decreases nitro-oxidative stress, mitochondrial dysfunction and hepatic ischaemia/reperfusion injury in mice. Rps. Pharm. Pharmacol. Rep. 2 (2), rqad013. 10.1093/rpsppr/rqad013 37092117 PMC10114105

[B62] Sanchez-Valle V. Chavez-Tapia N. C. Uribe M. Mendez-Sanchez N. (2012). Role of oxidative stress and molecular changes in liver fibrosis: a review. Curr. Med. Chem. 19 (28), 4850–4860. 10.2174/092986712803341520 22709007

[B63] Sato K. Marzioni M. Meng F. Francis H. Glaser S. Alpini G. (2019). Ductular reaction in liver diseases: pathological mechanisms and translational significances. Hepatology 69 (1), 420–430. 10.1002/hep.30150 30070383 PMC6324973

[B64] Scaglione S. Kliethermes S. Cao G. Shoham D. Durazo R. Luke A. (2015). The epidemiology of cirrhosis in the United States: a population-based study. J. Clin. Gastroenterol. 49 (8), 690–696. 10.1097/MCG.0000000000000208 25291348

[B65] Schnabl B. Choi Y. H. Olsen J. C. Hagedorn C. H. Brenner D. A. (2002). Immortal activated human hepatic stellate cells generated by ectopic telomerase expression. Lab. Invest. 82 (3), 323–333. 10.1038/labinvest.3780426 11896211

[B66] Shaker M. E. Ghani A. Shiha G. E. Ibrahim T. M. Mehal W. Z. (2013). Nilotinib induces apoptosis and autophagic cell death of activated hepatic stellate cells via inhibition of histone deacetylases. Biochim. Biophys. Acta 1833 (8), 1992–2003. 10.1016/j.bbamcr.2013.02.033 23499874 PMC5410771

[B67] Shao Y. Gao Z. Marks P. A. Jiang X. (2004). Apoptotic and autophagic cell death induced by histone deacetylase inhibitors. Proc. Natl. Acad. Sci. U. S. A. 101 (52), 18030–18035. 10.1073/pnas.0408345102 15596714 PMC539807

[B68] Shen S. Kozikowski A. P. (2016). Why hydroxamates may not Be the best histone deacetylase inhibitors--what some may have forgotten or would rather forget? ChemMedChem 11 (1), 15–21. 10.1002/cmdc.201500486 26603496 PMC4765907

[B69] Soliman A. M. Das S. Abd Ghafar N. Teoh S. L. (2018). Role of MicroRNA in proliferation phase of wound healing. Front. Genet. 9, 38. 10.3389/fgene.2018.00038 29491883 PMC5817091

[B70] Subedi P. Schneider M. Philipp J. Azimzadeh O. Metzger F. Moertl S. (2019). Comparison of methods to isolate proteins from extracellular vesicles for mass spectrometry-based proteomic analyses. Anal. Biochem. 584, 113390. 10.1016/j.ab.2019.113390 31401005

[B71] Suzuki H. Maruyama R. Yamamoto E. Kai M. (2013). Epigenetic alteration and microRNA dysregulation in cancer. Front. Genet. 4, 258. 10.3389/fgene.2013.00258 24348513 PMC3847369

[B72] Svegliati-Baroni G. Ridolfi F. Di Sario A. Saccomanno S. Bendia E. Benedetti A. (2001b). Intracellular signaling pathways involved in acetaldehyde-induced collagen and fibronectin gene expression in human hepatic stellate cells. Hepatology 33 (5), 1130–1140. 10.1053/jhep.2001.23788 11343241

[B73] Svegliati-Baroni G. Saccomanno S. van Goor H. Jansen P. Benedetti A. Moshage H. (2001a). Involvement of reactive oxygen species and nitric oxide radicals in activation and proliferation of rat hepatic stellate cells. Liver 21 (1), 1–12. 10.1034/j.1600-0676.2001.210101.x 11169066

[B74] Tao H. Shi K. H. Yang J. J. Huang C. Zhan H. Y. Li J. (2014). Histone deacetylases in cardiac fibrosis: current perspectives for therapy. Cell Signal 26 (3), 521–527. 10.1016/j.cellsig.2013.11.037 24321371

[B75] Tecalco-Cruz A. C. Rios-Lopez D. G. Vazquez-Victorio G. Rosales-Alvarez R. E. Macias-Silva M. (2018). Transcriptional cofactors Ski and SnoN are major regulators of the TGF-β/Smad signaling pathway in health and disease. Signal Transduct. Target Ther. 3, 15. 10.1038/s41392-018-0015-8 29892481 PMC5992185

[B76] Trams E. G. Symons A. M. (1957). Morphological and functional changes in the livers of rats after ligation or excision of the common bile duct. Am. J. Pathol. 33, 13–25. 13394688 PMC1934637

[B77] Trautwein C. Friedman S. L. Schuppan D. Pinzani M. (2015). Hepatic fibrosis: concept to treatment. J. Hepatol. 62 (1 Suppl. l), S15–S24. 10.1016/j.jhep.2015.02.039 25920084

[B78] Unsal V. Cicek M. Sabancilar I. (2021). Toxicity of carbon tetrachloride, free radicals and role of antioxidants. Rev. Environ. Health 36 (2), 279–295. 10.1515/reveh-2020-0048 32970608

[B79] Van Beneden K. Mannaerts I. Pauwels M. Van den Branden C. van Grunsven L. A. (2013). HDAC inhibitors in experimental liver and kidney fibrosis. Fibrogenes. Tissue Repair 6 (1), 1. 10.1186/1755-1536-6-1 PMC3564760 23281659

[B80] Wang B. Zhu X. Kim Y. Li J. Huang S. Saleem S. (2012). Histone deacetylase inhibition activates transcription factor Nrf2 and protects against cerebral ischemic damage. Free Radic. Biol. Med. 52 (5), 928–936. 10.1016/j.freeradbiomed.2011.12.006 22226832 PMC6010182

[B81] Wang N. Tan H. Y. Feng Y. G. Zhang C. Chen F. Feng Y. (2018). microRNA-23a in human cancer: its roles, mechanisms and therapeutic relevance. Cancers (Basel) 11 (1), 7. 10.3390/cancers11010007 30577536 PMC6356664

[B82] Wang Y. Stowe R. L. Pinello C. E. Tian G. Madoux F. Li D. (2015). Identification of histone deacetylase inhibitors with benzoylhydrazide scaffold that selectively inhibit class I histone deacetylases. Chem. Biol. 22 (2), 273–284. 10.1016/j.chembiol.2014.12.015 25699604 PMC4365786

[B83] Wimborne H. J. Takemoto K. Woster P. M. Rockey D. C. Lemasters J. J. Zhong Z. (2019). Aldehyde dehydrogenase-2 activation by Alda-1 decreases necrosis and fibrosis after bile duct ligation in mice. Free Radic. Biol. Med. 145, 136–145. 10.1016/j.freeradbiomed.2019.09.026 31557514 PMC6880805

[B84] Wu J. W. Krawitz A. R. Chai J. Li W. Zhang F. Luo K. (2002). Structural mechanism of Smad4 recognition by the nuclear oncoprotein Ski: insights on Ski-mediated repression of TGF-beta signaling. Cell 111 (3), 357–367. 10.1016/s0092-8674(02)01006-1 12419246

[B85] Xu F. Liu C. Zhou D. Zhang L. (2016). TGF-β/SMAD pathway and its regulation in hepatic fibrosis. J. Histochem Cytochem 64 (3), 157–167. 10.1369/0022155415627681 26747705 PMC4810800

[B86] Xu H. Sun F. Li X. Sun L. (2018). Down-regulation of miR-23a inhibits high glucose-induced EMT and renal fibrogenesis by up-regulation of SnoN. Hum. Cell 31 (1), 22–32. 10.1007/s13577-017-0180-z 28707079

[B87] Yoon S. Kang G. Eom G. H. (2019). HDAC inhibitors: therapeutic potential in fibrosis-associated human diseases. Int. J. Mol. Sci. 20 (6), 1329. 10.3390/ijms20061329 30884785 PMC6471162

[B88] Zeglinski M. R. Hnatowich M. Jassal D. S. Dixon I. M. (2015). SnoN as a novel negative regulator of TGF-β/Smad signaling: a target for tailoring organ fibrosis. Am. J. Physiol. Heart Circ. Physiol. 308 (2), H75–H82. 10.1152/ajpheart.00453.2014 25380815

[B89] Zhang C. Y. Liu S. Yang M. (2023). Treatment of liver fibrosis: past, current, and future. World J. Hepatol. 15 (6), 755–774. 10.4254/wjh.v15.i6.755 37397931 PMC10308286

[B90] Zhang Y. Wu L. Wang Y. Zhang M. Li L. Zhu D. (2012). Protective role of estrogen-induced miRNA-29 expression in carbon tetrachloride-induced mouse liver injury. J. Biol. Chem. 287 (18), 14851–14862. 10.1074/jbc.M111.314922 22393047 PMC3340269

[B91] Zhong Z. Froh M. Lehnert M. Schoonhoven R. Yang L. Lind H. (2003). Polyphenols from Camellia sinenesis attenuate experimental cholestasis-induced liver fibrosis in rats. Am. J. Physiol. Gastrointest. Liver Physiol. 285 (5), G1004–G1013. 10.1152/ajpgi.00008.2003 12791596

[B92] Zhong Z. Froh M. Wheeler M. D. Smutney O. Lehmann T. G. Thurman R. G. (2002). Viral gene delivery of superoxide dismutase attenuates experimental cholestasis-induced liver fibrosis in the rat. Gene Ther. 9 (3), 183–191. 10.1038/sj.gt.3301638 11859421

