# Supplementary material for: LP340, a novel histone deacetylase inhibitor, decreases liver injury and fibrosis in mice: role of oxidative stress and microRNA-23a
Source: Front Pharmacol. 2024 May 17;15:1386238. doi: 10.3389/fphar.2024.1386238 (PMC11140137; doi:10.3389/fphar.2024.1386238)

**Supporting Information**

**LP340, a novel histone deacetylase inhibitor, decreases liver injury and fibrosis in mice: role of oxidative stress and microRNA-23a**

Devadoss J. Samuvel^1^, John J. Lemasters^1,2^, C. James Chou^1,3^, and Zhi Zhong^1*^

^1^Department of Drug Discovery & Biomedical Sciences and ^2^Biochemistry & Molecular Biology, Medical University of South Carolina, Charleston, SC 29425 and ^3^Lydex Pharmaceuticals, Mt. Pleasant, SC 29464

Table of Contents

Table S1………………………………………………………………………………..Page 2-3

Figure S1 and legend………………………………………………………………….. Page 4

Figure S2 and legend…………………………………………………………………...Pages 5

| Table S1. Sources of Reagents | | |
| --- | --- | --- |
| *Items* | ***Sources*** | ***Catalog #*** |
| Acetylated histone-3 antibody (Lys9/14) | Santa Cruz Biotech., Santa Cruz, CA | SC-8655 |
| Acetylated tubulin antibody | Santa Cruz Biotech., Santa Cruz, CA | Sc-23950 |
| Alanine transaminase kit | Pointe Scientific, Uncoln Park, MI | A7526-150 |
| Chemiluminescence kit | Pierce Biotec., Rockford, IL | 34075 |
| Collagen 1 antibody | Abcam, Waltham,MA | ab260043 |
| Cytokeratin-19 antibody | Proteintech Group Inc, Rosemont, IL | 14965-1-AP |
| Diaminobenzidine substrate kit | Vector Lab, Newark, CA | SK-4100 |
| Dimethyl sulfoxide | Sigma-Aldrich, St. Louis, MO | D2650 |
| Direct Red 80 | Sigma-Aldrich, St. Louis, MO | 43665 |
| DMEM culture medium | Fisher Scientific, Waltham, MA | 11-995-065 |
| Fast green | Sigma-Aldrich, St. Louis, MO | F7258 |
| Glyceraldehyde 3-phosphate dehydrogenase antibody | Cell Signaling Technology, Danvers, MA | 2118 |
| Harris hematoxylin | Sigma-Aldrich, St. Louis, MO | HHS32 |
| 4-Hydroxynonenal antibody | Alpha Diagnostic, San Antonio, TX | 2118 |
| Hydroxypropyl methyl cellulose | Sigma-Aldrich, St. Louis, MO | H7509 |
| IQ-Sybr green mix | Bio-Rad Laboratories, Hercules, CA | 1708882 |
| Matrix metallopeptidase-9 antibody | Abcam, Waltham,MA | ab76003 |
| miRNeasy Micro Kit | Qiagen, Woburn, MA | 217084 |
| miRCURY LNA RT Kit | Qiagen, Woburn, MA | 339340 |
| Myeloperoxidase antibody | DAKO Corp., Carpinteria, CA | A0398 |
| 10% Neutral buffered formalin | Leica Biosystems, Buffalo grove, IL | 3800598 |
| Phospho-Smad2,3 antibody | Cell Signaling Technology, Danvers, MA | 8828 |
| Picric acid | Ricca Chemical company, Arlington, TX | 5860 |
| Pierce BCA protein assay kit | Pierce Biotec., Rockford, IL | PI23227 |
| Pierce Classic Immuno-precipitation Kit | Pierce Biotec., Rockford, IL | 26146 |
| Smad2, 3 antibody | Cell Signaling Technology, Danvers, MA | 8685 |
| Smad4 antibody | Santa Cruz Biotech., Santa Cruz, CA | Sc-7966 |
| α-Smooth muscle actin antibody | Cell Signaling Technology, Danvers, MA | 14968 |
| SnoN antibody | Santa Cruz Biotech., Santa Cruz, CA | Sc-9141 |
| Transforming growth factor-β1antibody | Santa Cruz Biotech., Santa Cruz, CA | sc-130348 |
| Tumor necrosis factor-α antibody | Cell Signaling Technology, Danvers, MA | 3707 |
| VECTASTAIN ABC HRP-kit | Vector Lab, Newark, CA | PK-4001 |
| YP00205631 –has-miR-23a-5P miRCURY PCR assay primer | Qiagen, Woburn, MA | 339306 |
| YP00203907 –U6snRNA miRCURY PCR assay primer | Qiagen, Woburn, MA | 339306 |

***Fig. S1. LP340 decreases ALT release, necrosis, and inflammation after CCl_4_ treatment in female mice.*** Female mice were injected with CCl_4_ (1:3 dilution in corn oil; 1.5 µl of the dilution/g mouse, *i.p*.) or an equal volume of corn oil once every 3 days for 6 wk. Mice received LP340 (0.05 mg/kg, ig, daily) or equal volume of vehicle (Veh) during the last 2 wk of CCl_4_ treatment. Blood and liver were collected after 6 wk of treatment. **A**, serum ALT; **, p<0.01 vs vehicle; ##, p<0.01 vs CCl_4_ without LP340. Data are means ± SEM (n = 3/group). **B-D**, representative images of histology H&E-stained liver sections (n = 3 per group). Bar is 50 µm.


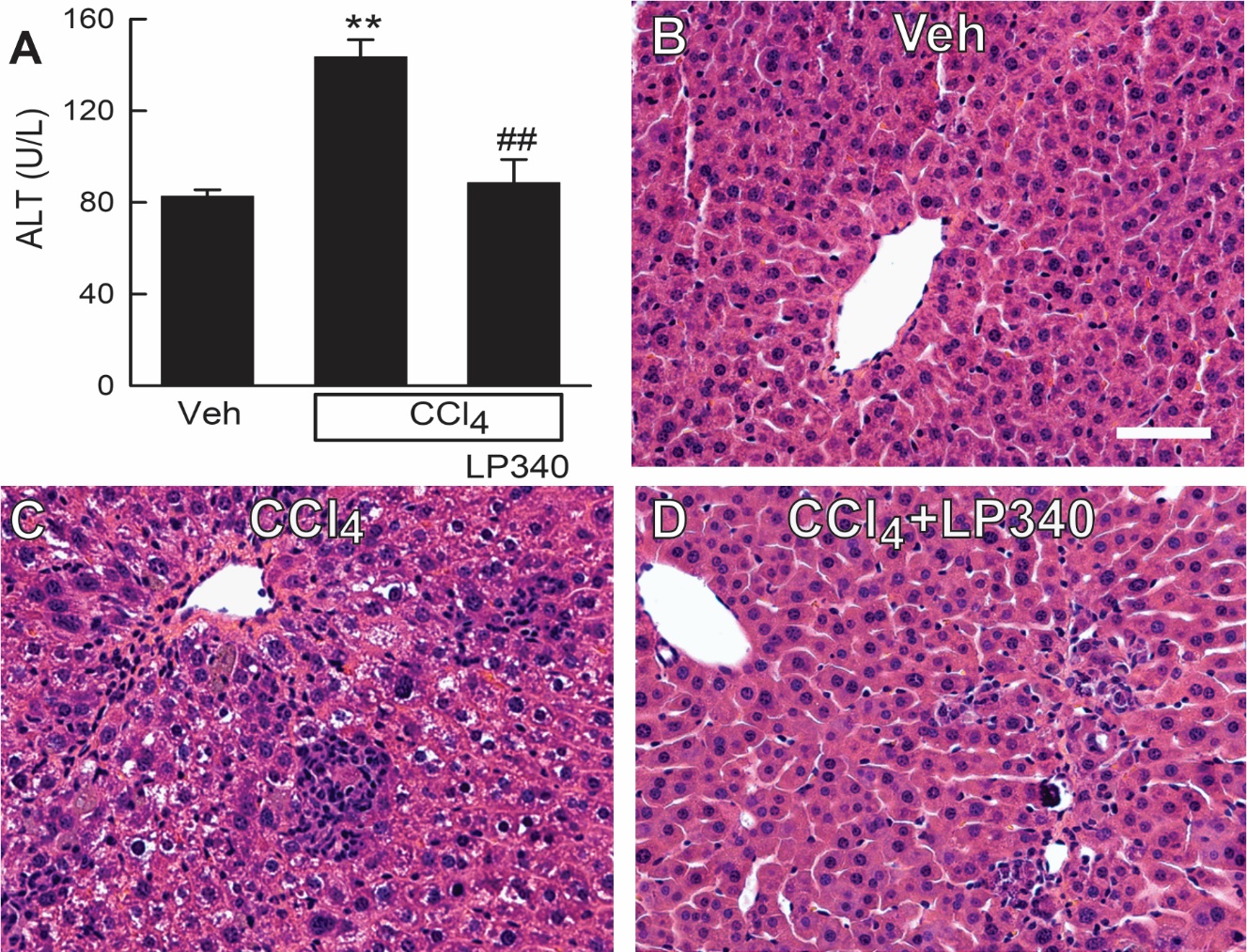


***Fig. S2. LP340 decreases liver fibrosis and TGFβ1 after CCl_4_ treatment in female mice.*** Female mice were treated, and livers were collected as described in Fig. S1. **A-C**, representative images of trichrome-stained liver sections. Bar is 100 µm. **D**, representative immunoblots for collagen-1 (Col-1), TGFβ1, and house-keeping protein GAPDH. **H-F**, quantification of Col-1 and TGFβ1 immunoblots by densitometry. **, p<0.01 vs vehicle (Veh); ##, p<0.01 vs CCl_4_ with LP340. Data are means ± SEM (n = 3/group).


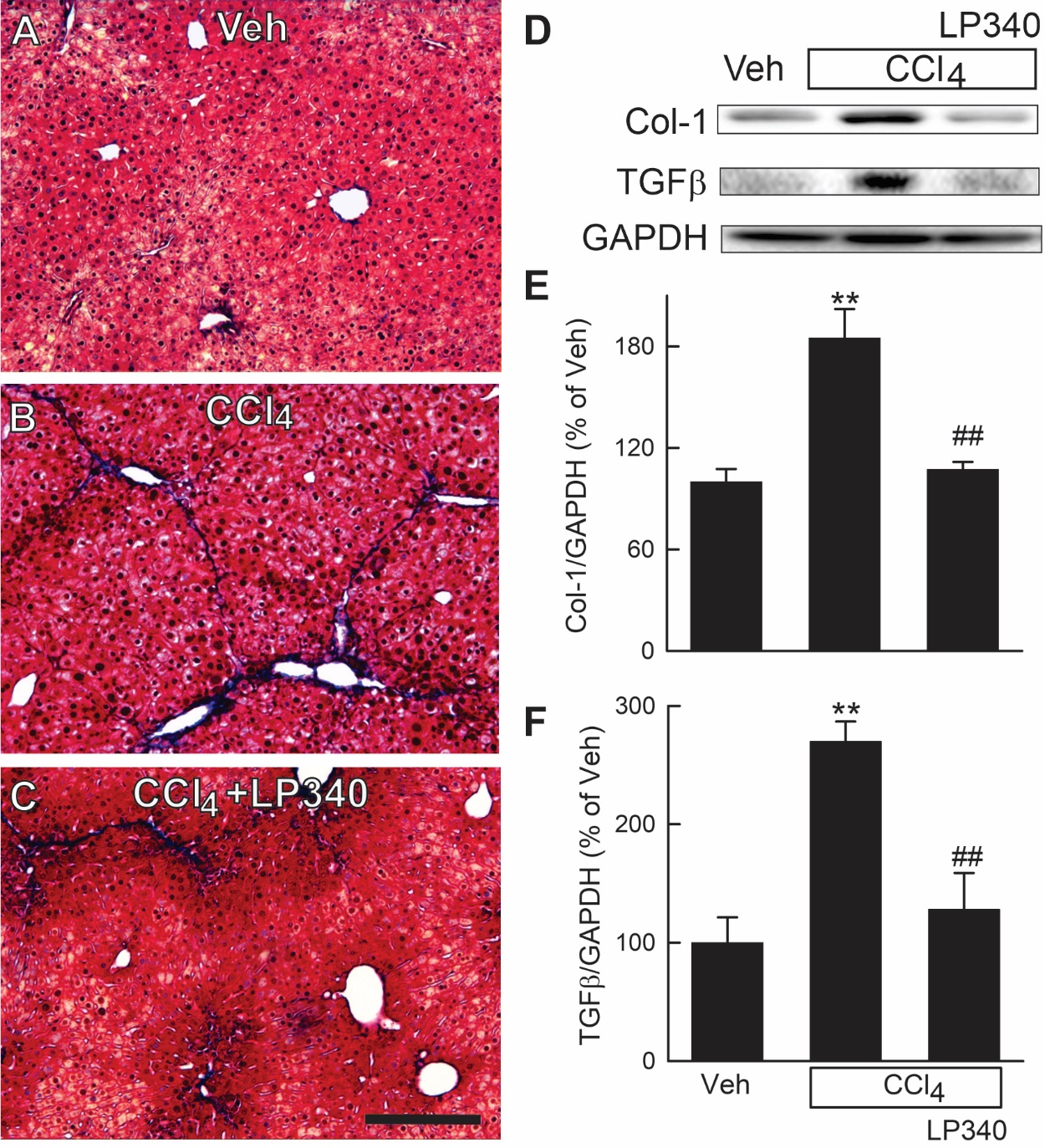

Supplement: Supplementary file 1 [file DataSheet1.docx]
